# Power consumption prediction for electric vehicle charging stations and forecasting income

**DOI:** 10.1038/s41598-024-56507-2

**Published:** 2024-03-18

**Authors:** K. C. Akshay, G. Hannah Grace, Kanimozhi Gunasekaran, Ravi Samikannu

**Affiliations:** 1 School of Advanced Sciences, Vellore Institute of Technology, Chennai, Tamil Nadu India; 2 Center for Smart Grid Technologies, School of Electrical Engineering, Vellore Institute of Technology, Chennai, Tamil Nadu India; 3 Botswana International University of Science and Technology, Palapye, Botswana

**Keywords:** Electric Vehicle infrastructure, Power consumption, Power scheduling, Forecasting, SARIMA, Mathematics and computing, Computer science, Electrical and electronic engineering

## Abstract

Electric vehicles **(**EVs) are the future of the automobile industry, as they produce zero emissions and address environmental and health concerns caused by traditional fuel-poared vehicles. As more people shift towards EVs, the demand for power consumption forecasting is increasing to manage the charging stations effectively. Predicting power consumption can help optimize operations, prevent grid overloading, and power outages, and assist companies in estimating the number of charging stations required to meet demand. The paper uses three time series models to predict the electricity demand for charging stations, and the SARIMA (Seasonal Auto Regressive Integrated Moving Average) model outperforms the ARMA (Auto Regressive Moving Average) and ARIMA (Auto Regressive Integrated Moving Average) models, with the least RMSE (Root Mean Squared Error), MAE (Mean Absolute Error) and MAPE (Mean Absolute Percentage Error) scores in forecasting power demand and revenue. The data used for validation consists of charging activities over a four-year period from public charging outlets in Colorado, six months of charging data from ChargeMOD's public charging terminals in Kerala, India. Power usage is also forecasted based on wheels of vehicles, and finally, a plan subscription data from the same source is utilized to anticipate income, that helps companies develop pricing strategies to maximize profits while remaining competitive. Utility firms and charging networks may use accurate power consumption forecasts for a variety of purposes, such as power scheduling and determining the expected energy requirements for charging stations. Ultimately, precise power consumption forecasting can assist in the effective planning and design of EV charging infrastructure. The main aim of this study is to create a good time series model which can estimate the electric vehicle charging stations usage of power and verify if the firm has a good income along with some accuracy measures. The results show that SARIMA model plays a vital role in providing us with accurate information. According to the data and study here, four wheelers use more power than two and three wheelers. Also, DC charging facility uses more electricity than AC charging stations. These results can be used to determine the cost to operate the EVs and its subscriptions.

## Introduction

The increasing adoption of electric vehicles (EVs) has led to a growing demand for charging infrastructure, particularly electric vehicle charging stations (EVCS). However, the operation and maintenance of EVCS require significant amounts of energy, which can result in high operating costs^1^. To address this issue, accurate prediction of power consumption is necessary to optimize the utilization of charging stations and minimize operational expenses. In addition, forecasting income is crucial for the sustainable and profitable operation of EVCS^2^.

Electric car adoption has been rising, and it is expected to keep on growing in the upcoming years. As a result, it is anticipated that there will be an increase in demand for EVCS, making efficient operation and maintenance of EVCS essential. Accurate prediction of power consumption and income can help optimize the utilization of charging stations, reduce operating costs, and improve revenue forecasting^3^. It can also aid in planning the charging infrastructure especially It can help with creating the charging pin for various vehicle kinds, including two-wheelers, three-wheelers, and four-wheelers. Additionally, it may be used to determine if clients favor AC or DC stations. Therefore, there is a significant motivation to develop an accurate and efficient model for power consumption prediction and income forecasting for EVCS. Time series models^4^ are considered a suitable approach as they can capture the trends and seasonality of power consumption accurately. The models of Autoregressive Moving Average (ARMA), Seasonal Autoregressive Integrated Moving Average (SARIMA) and Autoregressive Integrated Moving Average (ARIMA) are well recognized and often employed in time series analysis. These models are perfect for the job of estimating power consumption of EVCS and projecting income for the company since they have demonstrated promising results in properly forecasting electricity usage^5^.

This study's primary objective is to create a trustworthy time series model that can estimate EVCS's power usage and the firm's income with accuracy. The model will employ a variety of statistical techniques to find patterns and trends in the historical data of power usage and revenue. The company can optimize operations and efficiently manage resources by properly forecasting power use and income. This would not only save money but also contribute to improving the EVCS user experience for the consumers.

This paper is organized as follows: "Background" section presents the state of art literature review related to papers on EVCS and Forecasting the income based on prediction. "Methodology" section describes the methodology based on the time series models such as ARMA, ARIMA and SARIMA that are utilized to study data which helps in forecasting the future trends. Also, it gives a clear picture of the data collected from the charging stations of Colorado in USA. Preprocessing and Exploratory Data Analysis is done using packages in PYTHON. The results are discussed in “Results and discussions” section with graphical explanations. RMSE, MAE and MAPLE are the evaluation metrics used for the evaluation process. The forecasting plots shows that SARIMA model shows better result when compared with ARIMA and ARMA.

## Background

This journal paper, authored by^6^, presents a comprehensive conceptual framework for the successful integration of EVs into electric power systems. The framework encompasses two main domains: the technical operation of the grid and the electricity markets environment. The paper provides a detailed description of the various stakeholders involved in these processes and their respective activities. Additionally, several simulations are conducted to demonstrate the potential impacts and benefits that arise from the integration of EVs into the grid under this framework. These simulations include analyses of steady-state and dynamic behaviors^7^. The paper discussed three types of EVs that are currently relevant in the market: fully EVs, fuel cell EVs, and hybrid EVs. It acknowledges that the widespread adoption of these vehicles, particularly those relying solely on electric power, will have significant implications for the design and operation of electric power systems. In summary, this paper presents a comprehensive conceptual framework for integrating EVs into electric power systems, addressing technical and market aspects. It emphasizes the challenges and benefits of EV integration and proposes strategies for effective operation and management. The presented simulations provided insights into the potential impacts and highlight the importance of advanced charging control strategies and local-level control for optimal system performance.

This journal article, written by^8^, was centered on the creation of streamlined EV powertrain models for brand-new and current production cars, particularly the Tesla Roadster and Nissan Leaf. The models are based on published vehicle parameters and range information are compared with manufacturer specifications for range under various driving conditions and drive cycles. To validate the models, test results for the Tesla Roadster and Nissan Leaf are used, where a GPS-based smartphone app and Google Earth are used to simulate the geography of the test path. The paper demonstrated excellent correlations between the model projections, manufacturer data, and experimental outcomes. The study also considered the impacts of battery degradation over time and vehicle HVAC loads. These factors are considered to provide a more realistic assessment of the EV powertrain performance. Overall, the paper provides valuable insights into the performance of EV powertrains and their range capabilities.

Cai et al.^9^ discusses the energy issue and the state of ecosystem. According to them, the distribution network was facing new difficulties because of the growth of electric cars. The charging plan, the position and size planning for EV charging sites, and the cooperation coordination among EV and the distribution network all rely on the projection of charging demand and the scope of future EV growth. They assert that the growing size and charging capacity forecasting model for EV are established using the artificial neural network technique. The model's accuracy was demonstrated through an example of Kunming, a significant city in West China, and the projection of the size and filling capacity of EVs. It has been determined that their study offers a fresh approach to forecasting China's electric car market's future growth size and charging load.

The author^10^ addresses the problem of range anxiety and the variable anticipated range left in electric cars, which prevents their widespread adoption, in this article. The goal of the research is to comprehend the root causes of potential range prediction errors and how intelligent transportation systems (ITS) might assist in finding solutions. Eleven participants made 141 documented trips, and the findings showed that the range projected by the EV and provided to the driver was overstated by around 50% in comparison to the actual trip distance. According to the research, driving aggressively results in increased mistakes and has the most influence on range forecast accuracy. In summary, the paper reveals that range predictions in EVs are often overestimated compared to actual journey distance. Driving style and journey characteristics play significant roles in range prediction accuracy. The study suggests incorporating these factors into range prediction algorithms and utilizing intelligent systems to improve accuracy. It also highlights the importance of the driver's behavior in maximizing the available range of an EV.

The author^11^ sought to identify and measure links between the vehicle's kinematic parameters and its energy usage. To build the energy consumption calculation models, they used actual statistics on EV energy consumption. With the vehicle dynamics equation as the base physical model, they built three models using multiple linear regression. The input variables (predictors) used by each model are aggregated at a separate level, allowing prediction based on the input data available. One approach aggregates kinematic parameters in trips; another extends this model with fine-grained acceleration parameters over the journey; and the third uses fine-grained kinematic parameter values to forecast micro-trips energy consumption. Multiple linear regression (MLR) having a much higher correlation coefficient was shown to be a successful strategy for estimating energy usage using aggregated tour data by accounting for a significant percentage of the variability included in the data. The findings showed that increasing the degree of detail in a model has the potential to produce one that is more accurate. Finally, concluded as non-linear effects of regenerative braking are mapped by connecting traffic situations and driving style with CMF to create accurate models.

The author^12^ provides an overview of research on measuring and estimating energy consumption in EVs. The study emphasizes the significance of EVs in reducing oil dependence, improving efficiency, and mitigating carbon emissions. They developed a data collection system and analyzed 5 months of data to evaluate EV performance and driver behaviors. The findings revealed higher efficiency on in-city routes compared to freeways, with drivers adjusting behavior based on real-time energy usage information. The research explored the relationships between EV power and variables such as velocity, acceleration, and roadway grade, highlighting their impact on energy consumption. They proposed an empirical method and an analytical model for EV power estimation, successfully predicting instantaneous power and trip energy consumption. The study also demonstrated the feasibility of data collection and provides insights for enhancing EV energy efficiency. Limitations include a restricted dataset involving a single driver and vehicle, calling for broader studies with multiple drivers and commercially produced vehicles is required to validate the estimation model. Overall, this research contributes to understanding EV energy consumption and offers valuable insights for researchers and EV users.

In this article^13^, the difficulties in accurately estimating the energy consumption of EVs are discussed and potential solutions are provided. EVs are complex devices with multiple concurrent processes involving energy consumption and generation within different onboard systems. Achieving more precise simulations of energy consumption is crucial for gaining a better understanding of energy management in electric transport and ultimately contributing to a sustainable future with enhanced convenience in terms of distance range and recharging time. This paper addresses the problem of energy consumption simulation for EVs using various software packages and offers insights on making the simulation process more precise. By doing so, engineers can develop improved energy management strategies for EVs. Computer simulation plays a crucial role in the development and optimization of EV systems, and accurate modeling is a central challenge. Specific testing cycles such as NEDC, JC-08, and EPA cycle are utilized for powertrain simulation and optimization in different regions. In summary, this paper underscores the challenges of simulating energy consumption in EVs and proposes methods to enhance accuracy. It emphasizes the importance of precise modeling for improving energy management strategies, optimizing powertrain systems, and facilitating overall performance enhancement in EVs.

Several contrast methods^14^ using participatory sensing for predicting the individual energy (1) a comparison to the average using personalized customization; (2) two techniques to resemblance matching based on vehicle/driver/environment-dependent characteristics utilising pace profile comparison and driving habit match; and (3) an ensemble filtration strategy utilising matrix factorization. To find variables that depend on the driver, the car, and the surroundings, they also used a collaborative filtering technique based on matrix factorization and a black box framework. As there is a lack of systematic research on the effective use of the data for individualized prediction even though it can provide a variety of driving data.

Additionally, they conducted a case study using data from participatory sensing to forecast the distance to empty for electric vehicles, and they empirically assessed their results, that demonstrated that their approaches could greatly increase prediction accuracy. De Cauwer et al.^15^ to address the issue of range anxiety, they provided an energy consumption forecast method for EVs that was designed for energy-efficient routing. Using real-world measured driving data, geography data, and weather data, this data-driven technique anticipates consumption across every specific road in a road network.

They have approximated the energy consumption over road segments using a MLR models that links the energy consumption with minor driving characteristics and external factors. A neural network (NN) is utilized to forecast the unknowable microscopic driving parameters over a segment before departure based on the features of the road segment and the weather^16^. This method enables cost-optimization methods to choose energy-efficient paths and allows for the pre-departure forecast of every road in the system, there is energy usage.

The advantage of the data-driven method, according to their argument, is that the models can be easily modified in long run time to reflect changing circumstances. The development of a driving range estimation model for electric cars considering the influence of environmental temperature and driving circumstances is the main topic of this paper, written by^17^. Even though EVs are popular the limited energy density, high costs, and short cycle life of power batteries result in a restricted driving range compared to conventional vehicles. This research suggested a technique for estimating driving range that combines driving cycle recognition and prediction in order to overcome these difficulties. The driving cycle was first located using fuzzy C referring to clustering and Kernel principal component feature parameters. MATLAB/Simulink version R2023b^18^ is used to construct a fuzzy rule between the characteristic parameters and energy usage. The driving range estimation method is validated using a rotary drum test bench under the ECE 15 condition, and the results are compared with the estimation results of actual driving mileage. In summary, this paper presents a driving range estimation model for EVs that incorporates the effects of environmental temperature and driving conditions. The proposed method provided a new approach to estimate the driving range of EVs, considering various factors and optimizing energy consumption modeling.

The amount of energy needed for a future journey^19^ rely on a variety of elements, including driving style, knowledge of the topography of the road, the weather, and the flow of traffic. To anticipate the energy consumption for a future journey, Jiquan Wang, Igo Besselink, and Henk Nijmeijer discussed an algorithm that comprises of an offline algorithm and an online algorithm. They contend that the offline algorithm is intended to offer information for the driver to create future driving plans, whereas the online algorithm is intended to adjust the energy consumption prediction result based on current driving. They can confirm their energy consumption prediction algorithm with the aid of 30 driving tests.

The observed energy consumption for all trips was within the offline algorithm's allowable range, and the bulk of the differences between the measurement and nominal prediction was less than 10%, according to a comparison of the data^20^. Over the years, there has been a steady rise in the desire for electricity. A decent predictive model is, therefore, necessary to comprehend future consumption. Additionally, it aids in creating new plants and networks in order to prepare for anticipated increases in demand for electricity. Hence, the extensive use of ARIMA models for time series prediction by Praphula Jain, Waris, and Rajendra has produced encouraging findings. Using the ARIMA model, they have tried to predict how much energy will be used. After analyzing the electricity usage in IIT, (ISM) for the years 2004 to 2008, the seasonal ARIMA model was determined to be the best model. It was also able to predict consumption for the years 2008 and 2009.

Driving ranges^21^ of many EVs running on highways are projected by the authors, and this knowledge was utilized to develop a recommendation system. By using actual statistics on EV trips to build data-driven models, they can compute recommendations based on energy consumption. For well-known EV models, extremely precise prediction models are created using the authors' method. Prediction accuracy for new EV models, however, is lower than for popular EV models since fewer journeys by new EV models are made on the motorway than by well-known EV models. In order to solve this problem, the authors proposed a novel transfer learning strategy. A type of machine learning called transfer learning creates prediction models utilizing more than enough data on well-known EV models. They have put forth a novel transfer learning technique that incorporates more data to develop forecast models, more full information on popular EV models. Additionally, they tested their approach using real EV trip data. They claim that consequently, their approach had a prediction error rate that was 30\% lower than the traditional approach. Finally, it was found that the suggested method might be able to predict new EV models' energy consumption more precisely.

A Model for Predicting Electric Energy Consumption (EECP-CBL) that predicts electric energy consumption by combining a convolutional neural network (CNN) and a bi-directional long short-term memory (Bi-LSTM)^22^. Two CNNs are used in this study's initial module of the individual house electric power consumption dataset, that gathers the key information from a variety of sources. To create predictions, a Bi-LSTM module with two Bi-LSTM layers uses the data indicated above as well as time series patterns in both the forward and backward routes. They basically carried out these experiments to compare the forecasted results of the proposed model and the state-of-the-art models for the IHEPC dataset with various changes. Finally, findings showed that the EECP-CBL framework outperformed the most recent methods in terms of several performance measures for predicting electric energy usage. The efficacy of the electric energy usage prediction model is said to be enhanced in the future using various methods, including evolutionary algorithms.

This literature^23^ summarizes the research conducted by on the stability and power consumption of EVs using different modern control strategies. The study introduces a novel control approach based on Artificial Neural Networks (ANNs) to predict the yaw moment for ensuring the lateral stability of EVs with four in-wheel motors. Computer simulations are conducted to evaluate the robustness and power consumption of the proposed Neural Network Controller (NNC) compared to Sliding Mode Control (SMC). The results show that the NNC achieves stable motion near the driving limits with slightly lower power consumption compared to SMC. Additionally, the soft computing controllers exhibit robustness against system uncertainty and consume satisfactory energy from the electric motors. This research contributed to understand stability control in EVs and highlighted the potential benefits of employing modern control strategies to enhance stability and reduce power consumption.

Time series ML analysis is used for forecasting in many different industries. ML-driven data series analysis can help predict the following: Demand and sales. ML can help analyze historical data to predict customer demand or sales. In some cases, there may be a scarcity of historical time series data, making it challenging for ML models to learn meaningful patterns. In certain cases, time series data may lack clear patterns or trends, making it difficult for ML algorithms to identify meaningful relationships and make accurate predictions. Poor quality or inconsistent time series data can negatively impact the performance of ML models, leading to inaccurate predictions and unreliable insights. Certain industries or domains may have unique challenges that are not easily addressed by generic ML models.

This work summarizes the research on a novel ensemble method for forecasting EV power consumption in Spain^24^. The study addresses the challenges associated with increasing EV usage and the need for power companies to adapt their generation accordingly. The proposed approach combines ARIMA, GARCH, and PSF algorithms using ensemble learning to forecast EV power consumption. The non-stationary nature of the time series adds complexity, leading to the dynamic weighting of algorithms over time. The study demonstrated the effectiveness of the approach using the Weighted Absolute Percentage Error (WAPE) metric. The research contributes by developing an ensemble algorithm, analyzing non-stationary time series, applying the method to real data, and implementing it at the EV Control Center. Coefficients are periodically updated to handle evolving data, and future research could focus on optimizing update frequency and exploring specialized treatments for holidays and different charging station aggregations. Overall, this research advances the field of EV power consumption forecasting and showcases its practical applicability in the Spanish system.

To forecast the demand for charging new EV models with larger battery capacities, A DCFCS dynamic planning approach that considers user behaviour and probabilistic driving patterns was developed by Marjan Gjelaj and colleagues^25^. EVs (electric cars) seem to be a viable option for promoting green transportation and reducing CO2 emissions in urban areas. It is recommended to have saved charging need and synchronized charging need to lower the peak load from EVs and the expense of the charging infrastructure.

They considered a few configurations, such as synchronized storage feeding demand and charging demand, inside the stochastic planning technique. To reduce the running costs of the DCFCS and the peak demand for EVs, they have proposed an ideal BES as a replacement strategy. They also used setup strategies as a optimum multipurpose design issue with the primary objective of lowering grid-reinforcement costs. The last phase was to carry out an economic analysis to evaluate the technical and financial elements of DCFCSs, the life-cycle costs of BESs, and the financial performance of BES costs in relation to grid-reinforcement expenses. Predicting the charging demand of PEVs (the energy consumed during the charging session) could aid in the efficient management of the electric grid^26^. One of the key components of green buildings and microgrids is now energy usage tracking. A dataset with information on charging via public charging facilities in Nebraska, USA was used, over the course of seven years. They have also boldly used data from many stations to apply the predicted framework.

In that case, it is probable that those input variables have an even stronger link with consumption of energy, improving estimates for a smaller percentage of consumers. The same framework may be used with data from a smaller area or even just one station. The challenge they ran into in this study was trying to build a prediction model to explain charging and parking habits after analyzing a substantial amount of semi-random data. They assert that by examining the charging trends at both public and private charging stations, this analysis can be strengthened.

In this article, Kim and Kim^2^ examine the most recent EV energy usage models with the goal of offering recommendations for the development of EV apps in the future. They discuss EV energy consumption models in terms of modeling scale (microscopic vs. macroscopic), methodology, and they essentially divide the influencing aspects of EV energy consumption to four categories: vehicle dynamics, traffic, environment-related factors, and automobile component (data-driven as opposed to rule-based). Their investigation shows trends of rising macroscopic models that can be used to predict trip-level EV energy consumption, as well as growing data-driven algorithms that estimated EV consumption of energy via vast amounts of real-world information and machine learning techniques. And it is discovered that rule-based models predominate earlier literature, whereas data-driven EV energy usage estimation and its applications have been drawing growing study interest in the last few years. Finally, they concluded that multi-scale energy estimation models should be developed as a comprehensive modeling strategy and that energy estimation models appropriate for applications linked to vehicle-to-grid integration should also be developed.

Using many scaled geographic datasets over a 2-year period, Kim and Kim^27^ compare the forecasting approaches used to account for increases in power consumption brought on by the growing acceptance of EVs. They compared various modeling techniques based on historical data and exogenous variables, including long short-term memory (LSTM) modeling and Trigonometric, Box-Cox, auto-regressive moving average (ARMA), trend, and seasonality (TBATS) are other names for trigonometry exponentially smoothing state space. It was found that the historical data's importance was confirmed, and the effects of exogenous variables are evaluated on both macro and micro-scale geographic regions. When they compared time-series techniques with machine learning techniques, they found that machine learning had the advantage of requiring comparatively less model-fitting assumptions and straightforward hyperparameter tuning, but that it was not always highly predictive. They finally reached a decision. For macro data with generally straightforward patterns, the ARIMA model with regressors produced the best results.

With the help of other researchers, Straka et al.^28^ created a data-centric methodology to examine how the functions, behaviors, and attributes of the environment around slow-charging facilities affect how energy is distributed there. They examined the likelihood distribution of energy use and its relationship to indicators of charging occurrences to gain some fundamental knowledge. They gathered geospatial information and produced numerous potential features that modeled the physical environment in which the charging infrastructure functions. It was found and analyzed a relatively small subset of the most significant characteristics that are correlated with energy usage using statistical techniques. They distinguished the chosen characteristics by applying the approach to a particular class of charging infrastructure, as decided, for example, by the rollout strategy employed. The proximity of public venues, working residents, and business categories are associated with greater energy usage at carefully placed charging infrastructure. Age-related characteristics of the populace have an impact on how much energy is used at recharge stations that are installed according to demand. It was discovered that their work offered insightful information on the types of data to gather and incorporate into prediction models to help with the implementation of charging infrastructure and the planning of power networks.

A thorough breakdown of the many aspects of the EV industry and its charging system is provided by Nagaraju Dharavat, Naresh Kumar, and others^29^. Additionally, It provides a step-by-step procedure for implementing the Vehicle to Grid (V2G) concept, explains how artificial intelligence can be used to capture data from the EV battery, and analyses the costs and benefits of using the V2G method effectively. This article also includes a list of various EVs, storage locations, methods for charging EVs using DGs combined with EVCS, as well as a variety of other socio-technical concerns with EVs. The acceptance rate and current stage of EVs globally have received attention.

EVs are a desirable way to reduce rising fuel use and GHG emissions, but their widespread use may compromise the distribution system's reliability^30^. As a result, many methods are used to forecast the charging of EVs. Using a dataset made up of 2000 observations of charging events gathered from two public charging stations in Morocco, Mouaad Boulakhbar and colleagues compare the performance of four well-known deep learning models, namely ANN, recurrent neural networks (RNNs), LSTM, and gated recurrent units (GRUs), in predicting charging demand for EVs. The findings demonstrate that all four regression methods are capable of accurately predicting Morocco's demand for EV charging. They claim that these findings can both short-term ensure the grid's dependability and long-term direct the Moroccan National Office of Electricity and Water to create more charging stations. Mediouni et al.^31^ suggested a hybrid strategy to achieve this goal by accounting for factors like extra weight, road conditions, and driving habits. Physical and equation-based models are used to simulate the major EV parts. They primarily used a large synthetic dataset to illustrate various driving situations. A city car was also used to gather data from the real world. To connect mechanical and electric power, they created a machine-learning algorithm. In terms of R2 and root mean square error (RMSE), it turned out that the proposed models performed well. They also added that it might be useful for route planning by EV users to lessen range-anxiety, as well as for people making judgments regarding autos for the appropriate sizing of parts like the battery and powerplant.

This paper by Wang and Abdallah^32^ introduces the concept of a semi-decentralized robust network of electric vehicles (NoEV) integration system for efficient power management in a smart grid platform. The primary objective of this system is to tackle the challenge of balancing energy supply and demand, particularly in the face of fluctuating energy generated by renewable resources. To achieve this, the proposed approach integrates an aggregator with EV fleets using a blockchain framework. The system employs a multi-stage algorithm executed by the EVs, that incorporated a novel federated learning algorithm called Federated Learning for Qualified Local Model Selection (FL-QLMS) to predict power consumption accurately. The paper emphasized the significance of efficient distribution and utilization of renewable energy, highlighting the virtual power plant (VPP) as a vital intermediary in the smart grid ecosystem. NoEV addresses critical factors such as robustness, cost-efficiency of data storage, rapid response to demand, and scalability. In conclusion, this paper presented a sophisticated semi-decentralized system, leveraging blockchain technology and innovative federated learning algorithms, to integrate EVs into power management effectively. The NoEV system demonstrated improved efficiency, accuracy, and robustness in addressing the challenge of balancing energy supply and demand in the smart grid domain.

In order to meet the FCS's demand for electricity while reducing the cost of production and energy loss, energy management and optimization are consequently crucial for the mixed energy system^33^. A mixed-integer linear programming-based energy management technique was created as part of a model reference adaptive control to address this issue and enhance the charging station's performance. The results of modeling and testing the proposed system with the MATLAB/Simulink software are discussed. The evaluation finds that the recommended energy management system offers a performance that is optimum for the rapid charging station's integration with nuclear and renewable energy.

## Methodology

The need for power consumption forecasts is growing as more people switch to EVs so that charging stations can be efficiently managed^33^. In the end, accurate power consumption predictions can help with the efficient planning and construction of EV infrastructure.

**Figure 1:**
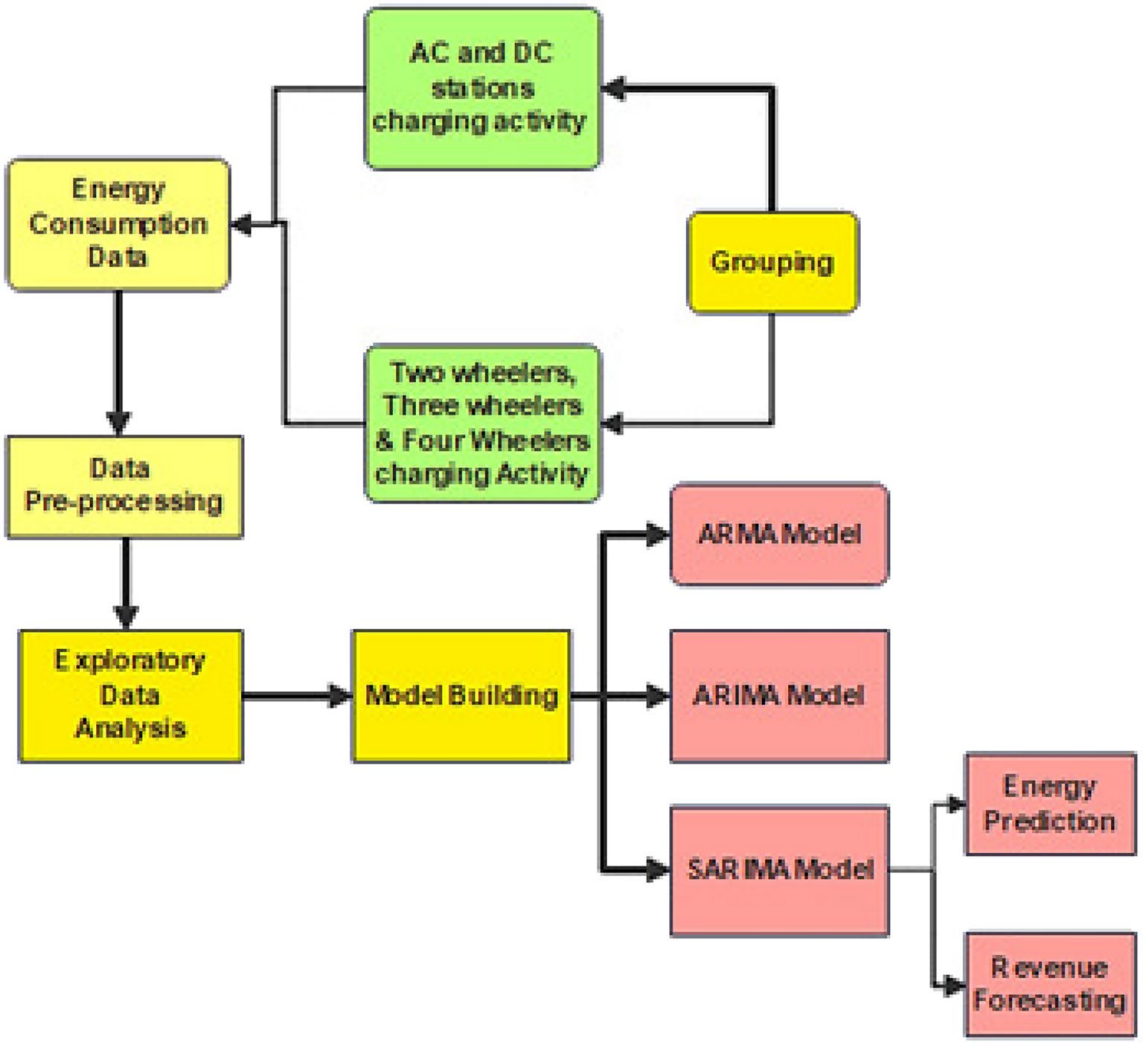
Architecture diagram.

A charging activity dataset downloaded from the Colorado GOVERNMENT website to predict future power consumption. The general architectural diagram for the energy prediction and revenue forecasting is shown in Fig. 1. Data collection, data preprocessing and exploratory data analysis are carried out. The project then acquired an additional charging activity dataset from chargeMOD for future usage prediction. However, since this dataset contained charging activity data for various types of stations and vehicles, the charging activity data was grouped into categories for AC stations, DC stations, two-wheelers, three-wheelers, and four-wheelers, and further data preprocessing and exploratory data analysis was performed. This classification allowed for more precise forecasting of power consumption based on the specific charging station and vehicle type.

### Model building

Time series models such as ARMA, ARIMA, and SARIMA are utilized to foresee future values based on historical data.

#### ARMA (autoregressive moving average) model

The ARMA model is made up of two parts: autoregression (AR) and moving average (MA). The present value of a series is modeled in an AR model as a linear combination of previous values of the same series^34^. The current value of a series is modeled in an MA model as a linear mixture of historical mistakes, which are the disparities between the series' actual and predicted values.

1 $$ARMA\left(p,q\right){:}\;{Y}_{t}=c+\sum_{i=1}^{p}{\varphi }_{i}{Y}_{t-i }+\sum_{i=1}^{q}{\theta }_{i}{\epsilon }_{t-i }+{\epsilon }_{t}$$

#### ARIMA (autoregressive integrated moving average) model

The ARIMA model is an extension of the ARMA model that differs the series to make it stationary before fitting an ARMA model^35^. The difference between consecutive values of the series is calculated until it becomes stationary.

2 $$ARIMA\left(p,q\right){:}\;{Y}_{t}=\alpha +{\beta }_{1}{Y}_{t-1}+{\beta }_{2}{Y}_{\left(t-2\right)}+\dots +{\beta }_{p}{Y}_{t-p}{\varepsilon }_{t}+{\phi }_{1}{\varepsilon }_{t-1}+{\phi }_{2}{\varepsilon }_{t-2}+\dots {\phi }_{q}{\varepsilon }_{t-q}$$

#### SARIMA (seasonal autoregressive integrated moving average) model

The SARIMA model is an extension of the ARIMA^35^ model that is used when the series contains a seasonal component that must be modeled. The SARIMA model adds seasonal parameters that capture the seasonal patterns in the data.

3 $$SARIMA\left(p,d,q\right){\left(P,D,Q\right)}_{m}$$

In summary, the ARMA model is used for stationary data, the ARIMA model for non-stationary data, and the SARIMA model for data having a seasonal component. These models can be used to estimate future values of a time series by estimating the model parameters and then utilizing them to forecast future values of the series.

### Identifying stationarity

To ascertain if a series has a consistent mean and variance over time, identifying stationarity is a fundamental step in time series forecasting. Visualizing the autocorrelation function (ACF) and partial autocorrelation function (PACF) plots is one method of determining stationarity^36^. The best models, such ARMA, ARIMA, or SARIMA are chosen for forecasting future values and increase the accuracy of our forecasts by determining stationarity in a time series^37^.

#### Data collection

The charging activity dataset is compiled from two sources, the first of which was a website that had data (refer Table1) on charging sessions in Colorado, USA, over a four-year period. Other dataset is taken from Kerala, India, from chargeMOD's public charging stations. The chargeMOD company provided the following datasets: a six-month overall dataset of charging activity, six-month charging activity datasets for AC and DC stations, nine-month charging activity datasets for two-wheelers, three-wheelers, four-wheelers and finally a subscription dataset to forecast revenue of the company. With this analysis which batteries are charged more frequently, charging trends of various types of automobiles can be determined and new subscription plans can be made.

**Table 1:** Colorado data (sample data set).

State province	Zip postal code	Start date/time	Start time zone	End date time	End time zone	Total duration	Charging time	Energy	GHG savings	Gasoline savings
Colorado	80302	1/20/2018 7:29	MDT	1/20/2018 9:22	MDT	1:52:59	1:52:41	4.608	1.936	0.578
Colorado	80302	1/20/2018 11:42	MDT	1/20/2018 15:27	MDT	3:45:39	2:22:15	8.786	3.69	1.103
Colorado	80301	1/20/2018 11:51	MDT	1/20/2018 12:54	MDT	1:03:01	1:02:50	4.94	2.075	0.62
Colorado	80302	1/20/2018 13:55	MDT	1/20/2018 13:57	MDT	0:02:00	0:00:00	0.0	0.0	0.0
Colorado	80305	1/20/2018 14:54	MDT	1/20/2018 15:52	MDT	0:58:33	0:58:25	3.023	1.27	0.379
Colorado	80301	1/20/2018 20:21	MDT	1/21/2018 8:06	MDT	11:44:35	3:09:24	8.738	3.67	1.097
Colorado	80305	1/21/2018 14:19	MDT	1/21/2018 16:12	MDT	1:53:38	1:53:27	11.336	4.761	1.423
Colorado	80301	1/21/2018 17:27	MDT	1/22/2018 7:41	MDT	14:14:38	4:53:09	19.976	8.39	2.507
Colorado	80302	1/22/2018 10:04	MDT	1/22/2018 16:15	MDT	6:11:13	2:06:43	6.62	2.78	0.831
Colorado	80302	1/22/2018 11:22	MDT	1/23/2018 9:41	MDT	22:18:26	2:14:06	4.792	2.013	0.601
Colorado	80302	1/22/2018 12:53	MDT	1/22/2018 14:47	MDT	1:53:19	1:53:05	6.899	2.898	0.866
Colorado	80304	1/22/2018 14:00	MDT	1/22/2018 15:19	MDT	1:19:03	1:18:47	4.776	2.006	0.599

#### Data description

The data from datasets from many sources are used to create the time series models. The Colorado dataset contains information about electric vehicle charging stations located in Colorado, USA. The main attributes of the dataset include the station name, zip code, start date, and end date of the charging session, total duration of the session, and energy consumption in kWh. The dataset provides insights into the usage patterns and environmental impact of EV charging in Colorado. To predict or forecast future charging behavior, the datasets are split into a training set and testing set such that 70% of the data is for training, while the remaining 30% is for testing the predictive models. Subsequently training different models on the training set, the performance of each model is evaluated on the testing set to determine which model performs the best. The model with the highest accuracy is selected and used for future predictions of charging behavior.

The overall charging activity dataset contains data on the charging activity of users at charging stations, including user id, station id, station type, start date, stop date, total duration, and energy usage in watt-hours (wh). The charging activity dataset is further broken down by vehicle type, including AC usage, DC usage, two-wheelers, three-wheelers, and four-wheelers. Each dataset has the same attributes as the overall charging activity dataset. Finally, the plan subscription dataset contains information about user subscriptions, including user id, plan id, plan name, plan price, validity, plan energy, and balance. For time series forecasting, only the date and usage attributes are used, while other attributes are used for exploratory data analysis.

#### Data preprocessing

Initially charging activity dataset from Colorado is downloaded and null values referred as missing values denotes the absence of data for a certain observation or variable. Numerous things, including errors in data collection or entry, problems with the equipment, or simply the absence of data points, can result in null results. Since null values can impair the accuracy of the analysis and predictions, dealing with them is a crucial part of data preparation. Hence the rows containing the null values are removed. The only important fields needed for time series forecasting are the date field and usage field. That is locating and eliminating any duplicate or unnecessary variables, such as variables unrelated to consumption or pricing data, that are not required for the time series analysis. By concentrating on the pertinent variables that are connected to the time series pattern, this approach is crucial for time series forecasting since it reduces noise and increases the model's accuracy.

The dataset contained daily timestamps corresponding to the usage, so the data was converted into monthly energy usage and used for time series forecasting. Through the process of aggregating or summarizing the data points for each day, the minute-by-minute data are grouped into daily data. Depending on the needs of the study, this can be accomplished by calculating the mean, median, or total of the minute-by-minute consumption data for each day. For data aggregation, mean for the minute-by-minute consumption data for each day is used. This procedure is crucial for time series forecasting as it aids in reducing data noise and unpredictability and offers a more comprehensive daily perspective of the time series pattern, making it simpler to identify seasonality and long-term patterns.

The next step is date-time manipulation, that includes changing the date and time information into a format appropriate for time series analysis or extracting certain properties like the day of the week or the month of the year. This procedure is crucial for time series forecasting because it helps the models more precisely identify patterns and trends in the data and produce forecasts for future values. If the data was found to be non-stationary, it is made stationary by first-order differencing. The data is then used to get ACF and PACF plots to determine the order of the AR and MA parts in different time series models. The second dataset is collected from the company chargeMOD and contained charging sessions of different users. Since the charging activity dataset is further grouped for AC and DC stations and two, three, and four-wheeler charging session data. Finally, a subscription dataset from the same company is used to predict revenue through different subscription plans using the same steps, excluding splitting it according to station type and vehicle type.

Overall, the data preprocessing steps involved cleaning the data, converting it into monthly or daily usage, checking for stationarity, and using ACF and PACF plots to determine the order of AR and MA parts in time series models. The dataset was also split based on station type and vehicle types to get a more accurate prediction. In time series forecasting, splitting a dataset entail separating the data into two parts: a training set and a testing set. The time series model is created using the training data, and its effectiveness is assessed using the testing data by contrasting the projected values with the actual values. The sequence of the data points is crucial for time series analysis; hence attention should be used while dividing the dataset. A rolling window technique is a popular method for dividing a time series dataset, with the training set consisting of the first portion of the time series and the testing set consisting of the second half of the time series. The dataset is divided into a training set, which contained 70% of the data, and a testing set, that contained the remaining 30%, to create our time series forecasting model.

#### Autocorrelation function (ACF)

**Figure 2:**
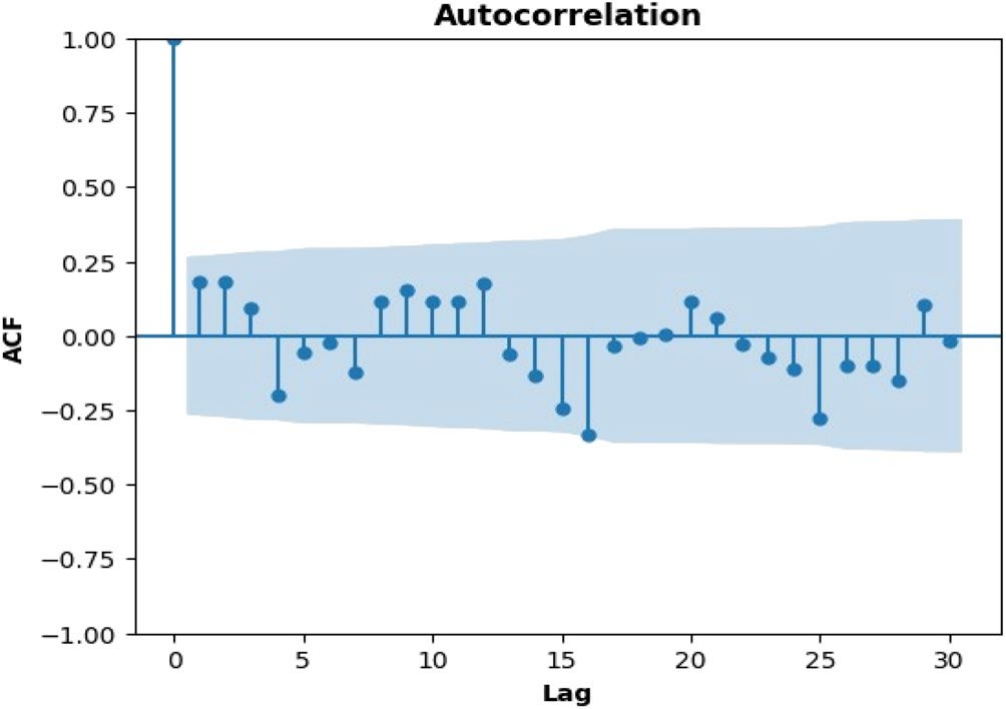
Autocorrelation function (ACF).

Order for the MA model is taken as 0 since there is no line extending in the region as shown in Fig. 2. The Autocorrelation Function, or ACF, is a statistical tool for determining the degree of correlation between a time series and its lagged values^38^. By plotting the autocorrelation values against the corresponding lags, the ACF is represented graphically in the ACF plot. The existence of a pattern in the data that repeats every k time step is implied by a substantial autocorrelation at lag k, which suggests that the value of the time series at time t is strongly correlated with its value at time t-k. An absence or mild autocorrelation at lag k indicates that the data do not have such a repeating pattern. Indicators indicating the type of process creating the time series, such as the existence of seasonality or trends, can also be found in the shape of the ACF plot.

#### Partial autocorrelation function (PACF)

**Figure 3:**
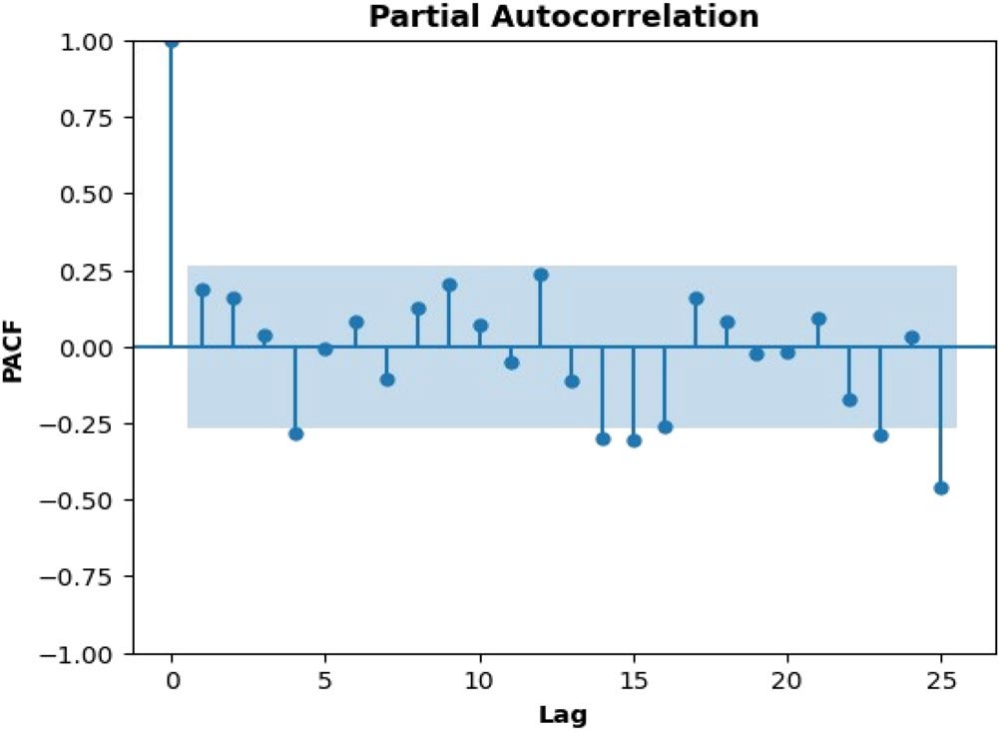
Partial autocorrelation function (PACF).

Order for the AR model in Fig. 3 is taken as 5, as only few lines are extending the region. Another tool used in time series analysis to comprehend the correlation structure of a time series is the partial autocorrelation function (PACF) graph. The correlation between a time series and its lags is displayed using a PACF plot while accounting for the impact of shorter lags. After accounting for all shorter delays (i.e., 1 through k-1), the PACF at lag k is the correlation between the time series at time t and its lagged value at time t-k. The order of an autoregressive (AR) process can be determined using a PACF display. The order of a moving average (MA) process can be determined using a PACF graphic. For an MA process, the PACF will specifically degrade gradually. A stationary time series is one in which the correlation coefficients rapidly decrease as the lag lengthens, but the PACF plot abruptly ends after a predetermined number of lags. In contrast, a non-stationary time series will show ACF and PACF plots that are progressively decaying, which suggests the presence of a trend or seasonality.

#### Evaluation metrics

RMSE, MAE, and MAPE scores are the three common assessment metrics to compare the performance of various time series models. These measurements are frequently used to evaluate a predictive model's ability to match the data and predict the future accurately.

Using these assessment measures, the performance of three distinct models—ARMA, ARIMA, and SARIMA—on various datasets are assessed. As indicated by its reduced RMSE, MAE, and MAPE scores, our study demonstrated that the SARIMA model consistently outperformed the other models across all datasets. This suggests that the SARIMA model is more adept at identifying patterns and trends in the data and producing precise forecasts of future values. These findings led us to choose the SARIMA model as the preferred model for forecasting the future. When compared to the ARMA and ARIMA models, the SARIMA model performed better, giving it a solid option for predicting future values. Overall, the assessment metrics gave us useful information about the advantages and disadvantages of the various models, enabling us to choose the best model for future projections.

After analyzing the Colorado EV charging activity dataset, it is found that some charging stations had significantly higher power consumption compared to others, indicating that factors like location and charging infrastructure play a significant role in energy consumption, also found that the power consumption is higher during weekdays compared to weekends, which could be due to increased usage by commuters. Overall, these insights can help in developing the necessary electrical infrastructure and evaluating the cost of operating EVs. Additionally, these findings can be used to optimize the placement of charging stations based on factors like vehicle type and location.

### Software’s and packages used

Users can generate interactive dashboards and reports using the business intelligence and data visualization software Tableau Desktop^39^. scipy package in Python offers various statistical methods like hypothesis testing, probability distributions, correlation analysis, and many others. Additionally, statsmodels, NumPy, in pandas package and datetime package in Python are used for statistical modeling, data analysis, manipulation and for handling dates and timings.

### Exploratory data analysis

Exploratory data analysis (EDA) and the visualization of large, complicated datasets are two common uses for the potent tool Tableau. Analysts may visualize and examine data in several ways with the help of the software's capabilities and features. Tableau uses a variety of plots to convey data visually, including bar charts, pie charts, and line graphs. When it comes to seeing significant patterns and trends in the dataset, such as seasonal patterns, outliers, and relationships between variables, these visualizations are very helpful. Using Tableau, analysts can rapidly and simply analyze huge datasets, produce insights, and present their results to stakeholders in a way that is both concise and effective.

**Figure. 4:**
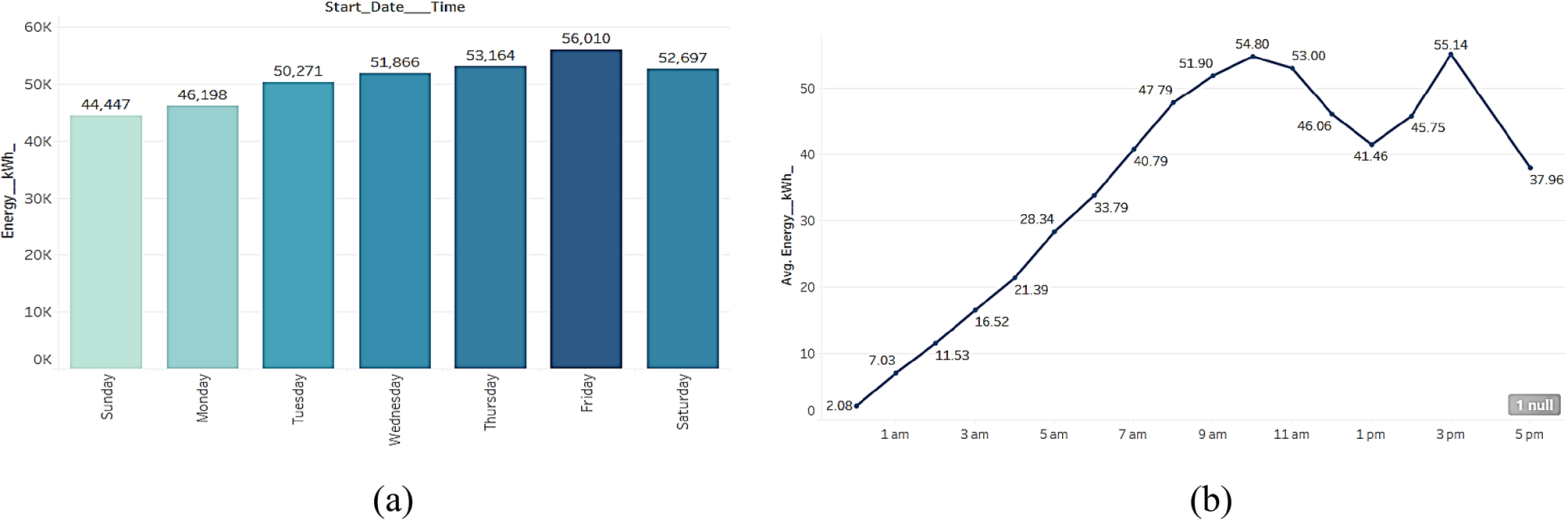
Average energy consumed over weekdays and each hour (**a**) Change in energy consumption over weekdays and (**b**) average energy consumed in each hour.

**Figure.5:**
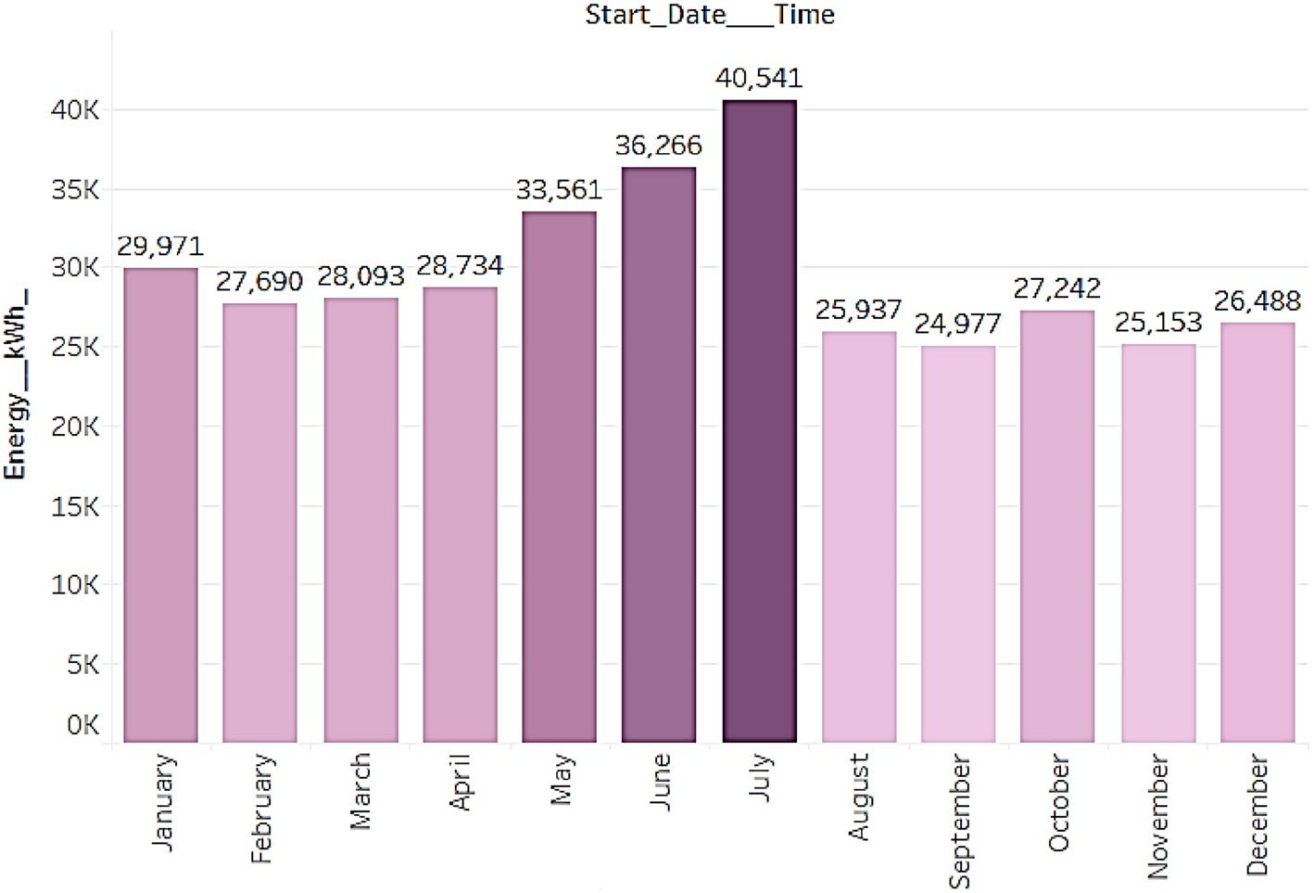
Change in energy consumption over months.

Friday of every week consumes maximum energy of 56,010 units as can be clearly seen in Fig. 4a. Perhaps people prefer to charge their EVs on Fridays, or it could be due to other factors that result in higher energy consumption on Fridays. There are specific periods of the day when energy usage is at its peak, according to an hourly examination of consumption statistics. The data specifically reveals that 10 am and 3 pm are the times when energy use is at its highest as seen from Fig. 4b. Compared to other times of the day, there is a noticeable increase in the quantity of energy utilised during these periods. To optimise energy distribution and supply at peak times or to inform policies that encourage energy saving during these hours, this information can be helpful in a variety of ways. Businesses and consumers can benefit from significant insights that can be gained from knowing when and where energy is being used. This will allow them to make educated choices about their energy consumption and perhaps cut expenses. The month-wise analysis from Fig. 5, shows that July has maximum energy usage of 40,541 units followed by June and May. And the lowest consumption is recorded in the month of September.

#### Charging activity dataset

**Figure.6:**
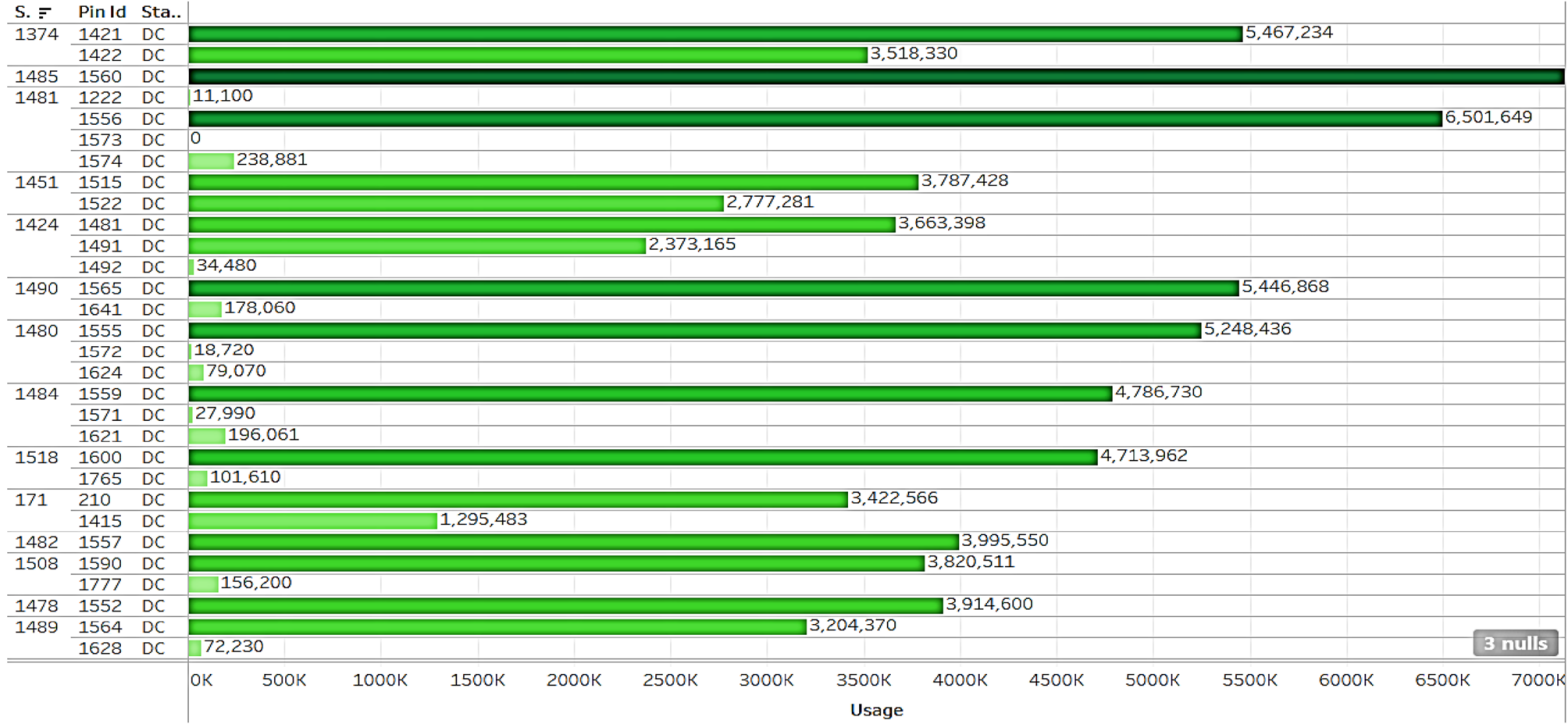
Usage classified into station ID, pin ID, station type.

*T*he charging stations are located at different places in the city*.* Also, the type of charging station, number of vehicles used and type of vehicle changes as per the location. In order to identify the type of station and the type of vehicle, station IDs are provided with Pin Id*.* For analysis, the station ID 1490 and Gun ID 1641, 1565 is considered. The Station Id 1490, Gun with Id 1641 had comparatively very less usage compared to Gun with Id 1565 as can be seen from Fig. 6. The limited or nonexistent utilization of these specific charging stations suggests that several variables may be at play. One explanation can be the little parking available for larger vehicles. Another reason can be that the charging pins have some problems. Additionally, it's probable that there aren't many people that use electric vehicles nearby, which results in little to no utilization. From the plots, it can be inferred that other than manual stop, power failure has become the second most reason for stopping from charging for AC charging stations, and in DC stations it is maximum remotely stopped. It is found 1,431 times charging stations are force stopped in AC stations whereas only 92 times in DC stations.

**Figure 7:**
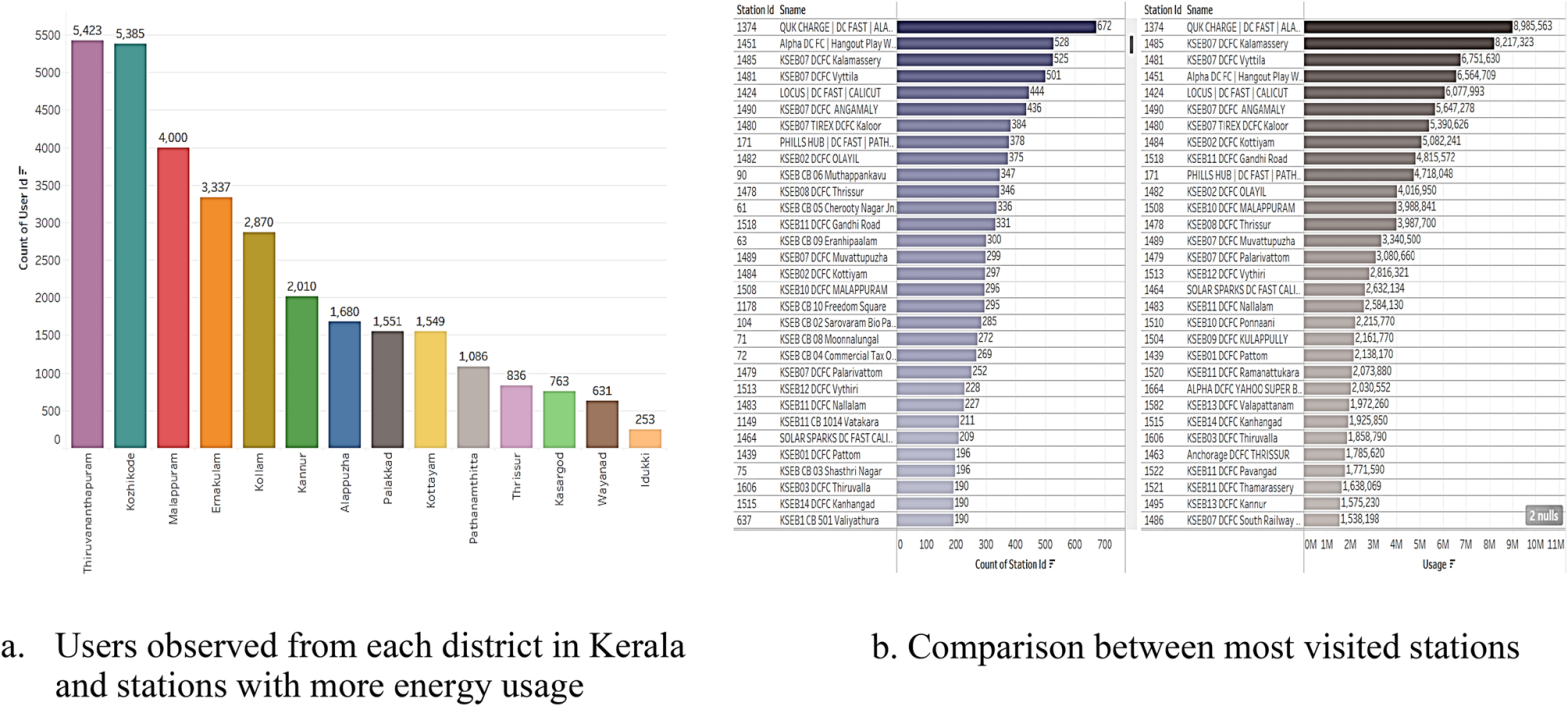
Users in each district and most visited stations.

Figure 7a depicts the users count in Kerala from each district and Fig. 7b portrays DC fast charge Alappuzha charging station turn to be the top visited station 672 times followed by Alpha DC FC 528 times. Maximum power consumption is also in DC fast charge Alappuzha with 8985.6 (kWh) followed by DC FC Kalamassery with 8217.3 (kWh). Thus, these findings imply that to alleviate the crowding issues in these charging stations, installing more charging pins could be a feasible solution, as the data indicates a higher number of charging sessions are recorded in these specific locations.

#### Plan subscription dataset

**Figure.8:**
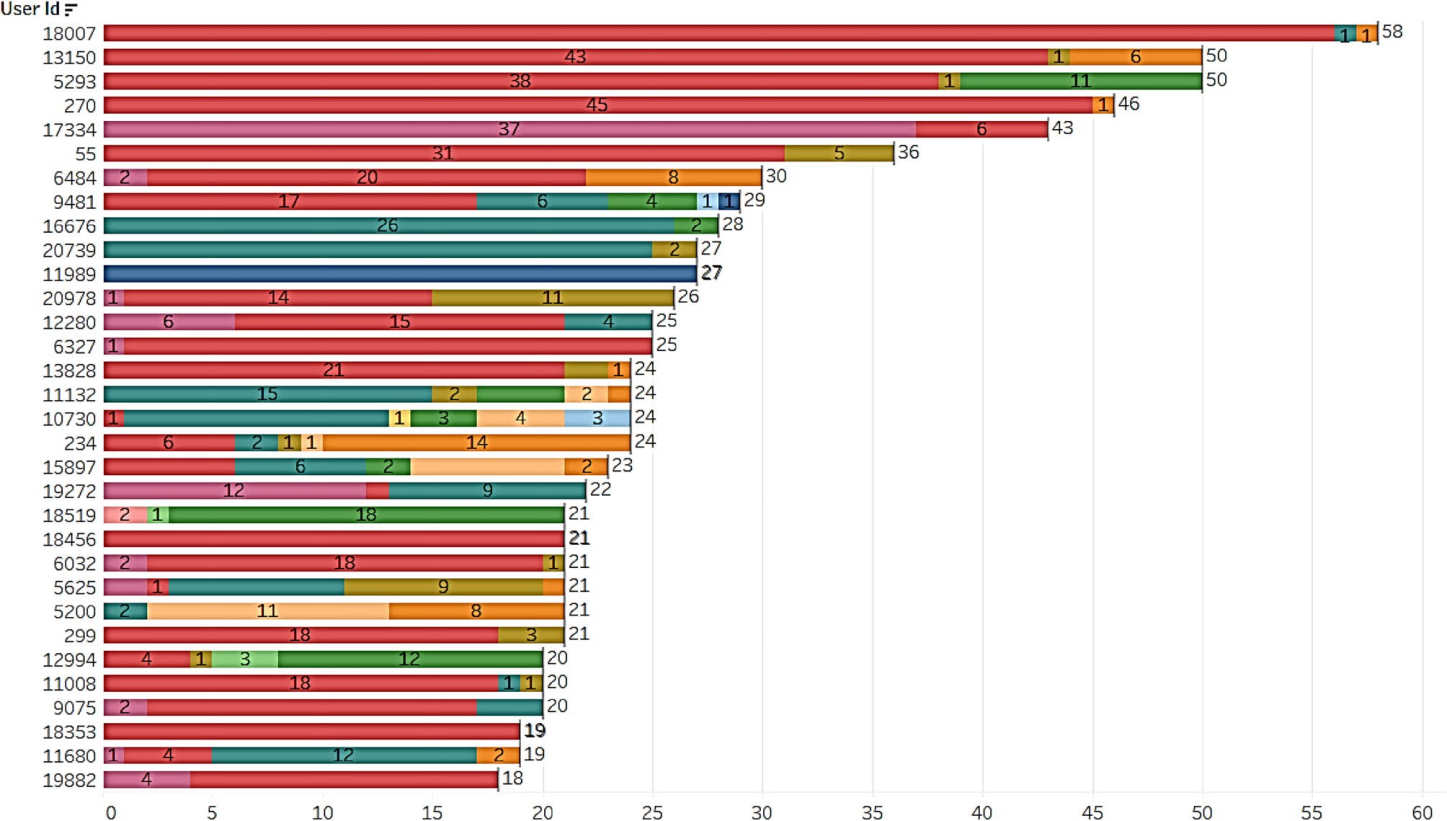
Users and total plans used.

**Figure 9:**
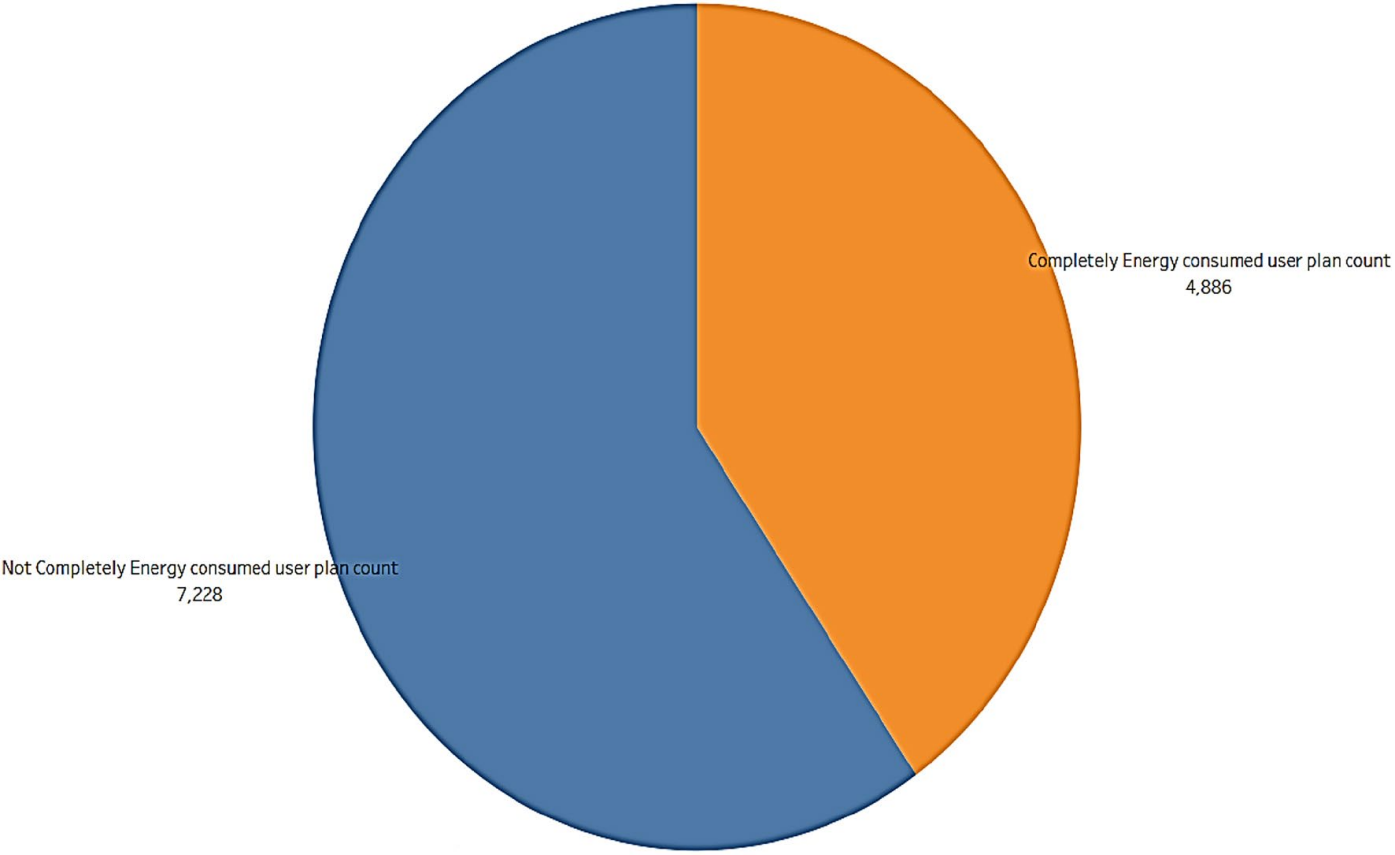
Plans used before their expiry date.

**Figure. 10:**
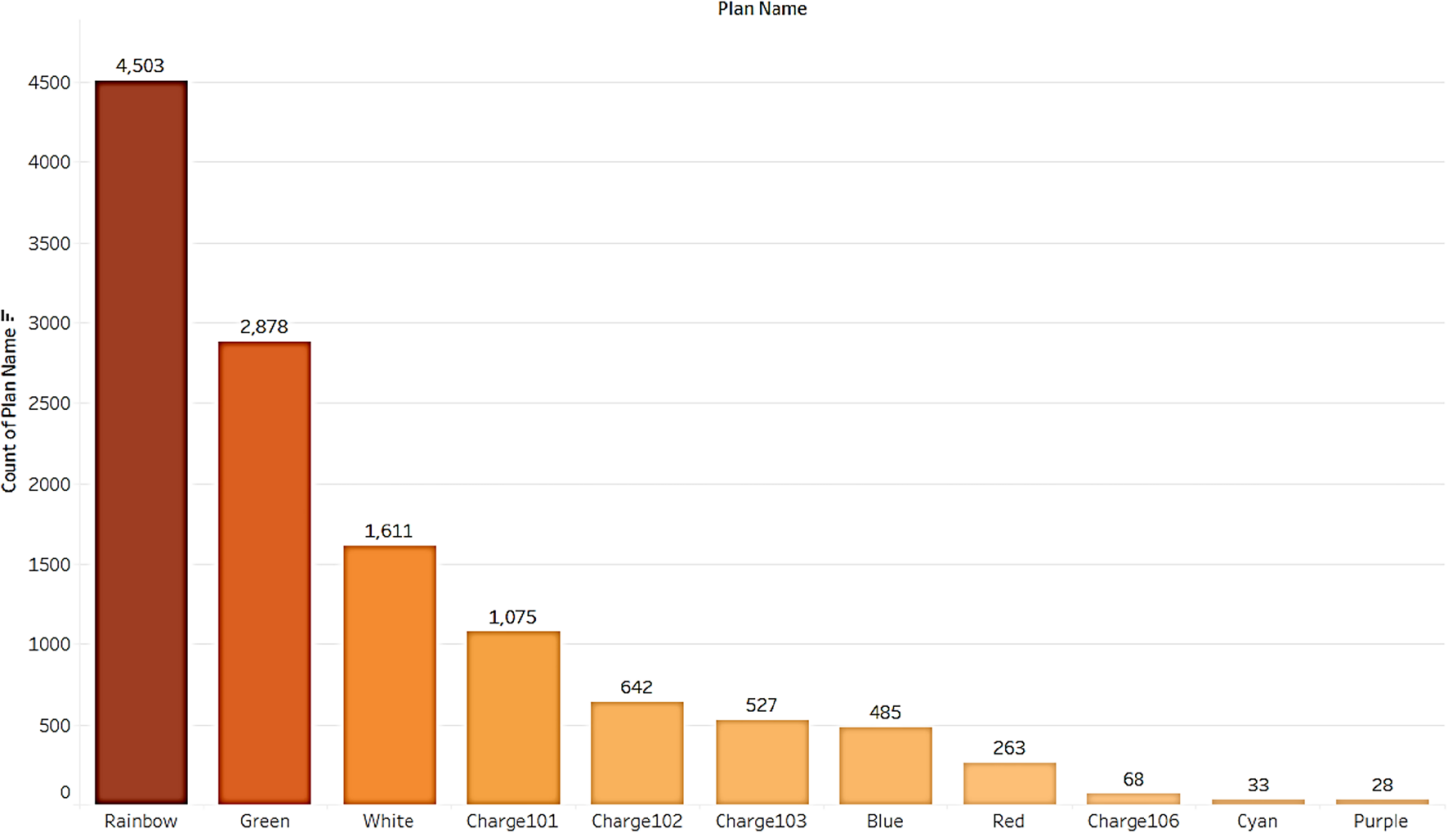
Top plans executed.

**Figure 11:**
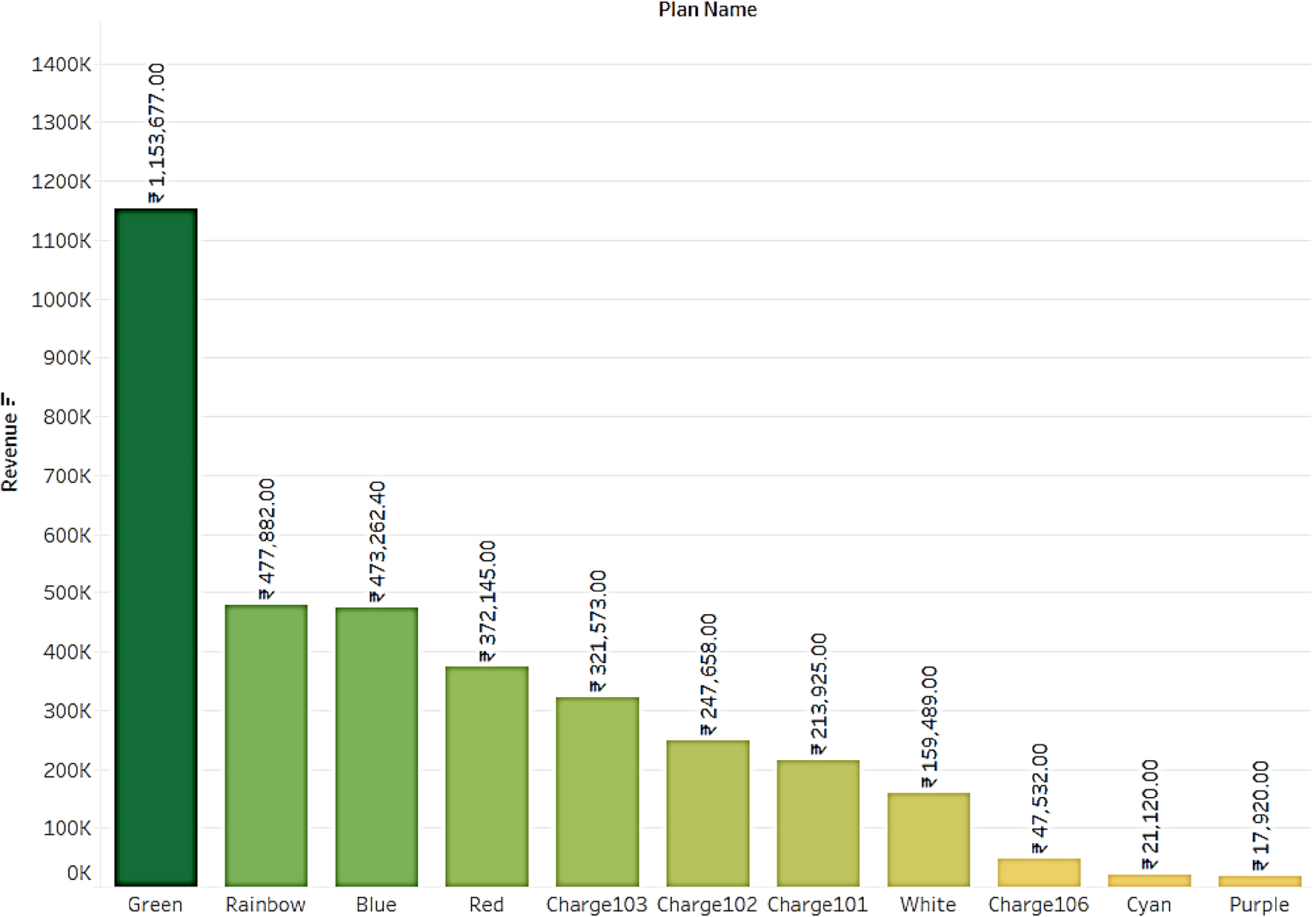
Total revenue from each plan.

The users and total balance in their plans are calculated and found 5% people only completely used the plans before expiry. 8% users had more than 50 units in their subscription plan as portrayed in Fig. 8. It can be inferred that more than 50% of the users are not completely using their subscription plan from Fig. 9, which contributes to the company's revenue. Figure 10, displays users and the total plans of each user are analyzed. The top 3 plans executed by users are Rainbow, Green, and White with a count of 4503, 2878, and 1611. From this, it can be concluded that altering Plan Rainbow and Plan Green may result in a rise in income creation, particularly through these plans. The plan Green generated revenue of Rs. 11,53,677 followed by Rainbow with Rs. 4,77,882 and Blue with Rs. 4,73,262. Revenue generated per day is analyzed and found to be increasing over days even though a dip is found at some points as seen from Fig. 11.

## Results and discussions

### Forecasting plots of COLORADO dataset

**Figure 12:**
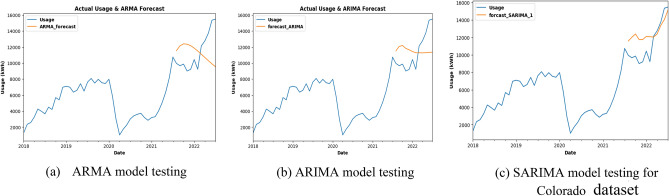
Energy usage forecasted using ARMA, ARIMA and SARIMA for 1 year.

The Energy usage is forecasted using the ARMA model for the last 1 year for already existing data can be clearly seen from Fig. 12a. and b shows that energy usage is forecasted using the ARIMA model for the last 1 year for already existing data. In Fig. 12c, energy usage is forecasted using the SARIMA model for the last 1 year for already existing data. The ARMA model was trained and tested, and it was found that the predictions are off, with the graph showing a downward trend rather than the anticipated increasing trend. Table 2 gives a summary of accuracy measures used for colarado dataset. The scores for Root mean squared error (RMSE), MAE, and MAPE are 3227.79, 2885.26, and 0.25, respectively. As opposed to the ARMA model, the ARIMA model produced more accurate predictions after evaluating the data, with lower RMSE, MAE, and MAPE scores.

**Table 2:** Summary: evaluation metrics for Colorado dataset:

MODEL	ARMA	ARIMA	SARIMA
RSME	3227.79	3022.34	1869.13
MAE	2885.26	2484.33	1572
MAPE	0.25	0.22	0.16

**Figure 13:**
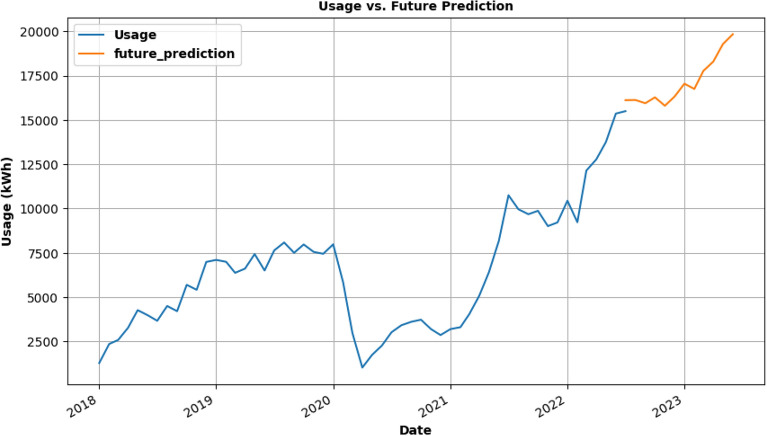
Future prediction using SARIMA model for 1 year.

However, testing with the SARIMA model produced the most encouraging findings since it is very accurate and correctly caught the increasing trend. This model's RMSE, MAE, and MAPE scores are much lower than those from the other models (1869.13, 1572, and 0.16, respectively). These findings led to the determination that the SARIMA model is appropriate for predicting future energy use. By the middle of 2023, Colorado's electricity consumption is anticipated to reach 20,903 units, according to model forecasts. Energy consumption trends can be forecasted using the SARIMA model and the knowledge from this study, that will ultimately result in more effective and sustainable energy use. In Fig. 13, SARIMA model is being used for future usage consumption prediction of the COLORADO dataset for 1 year.

### Forecasting plots of charging activity dataset

After the ARMA model had been trained and tested using the company's Charging Activity dataset, it is discovered that the forecast is inaccurate and had missed the trend, producing a graph with a straight line as a consequence. The results are 641.96, 520.64, and 0.23 for RMSE, MAE, and MAPE, respectively. However, there is a minor gain in accuracy when using the ARIMA model, although it too exhibited a straight-line graph. Although the trend is somewhat caught, the RMSE, MAE, and MAPE scores are lower than the ARMA model. The SARIMA model, with a graph that showed an upward trend, is examined and shown to have higher prediction accuracy than both the ARMA and ARIMA models. The scores obtained from the other models are higher than the RMSE, MAE, and MAPE scores, which are 539.37, 268.06, and 0.12, respectively. The SARIMA model is chosen to anticipate future energy consumption, and it is predicted that the company's power consumption will be around 3637 Wh on March 1, 2023. A summary of the evaluation metrics for the charging data set is given in Table 3.

**Table 3:** Summary: evaluation metrics for charging data set.

MODEL	ARMA	ARIMA	SARIMA
RSME	641.96	600.66	539.37
MAE	520.64	484.10	268.06
MAPE	0.23	0.22	0.12

**Figure.14:**
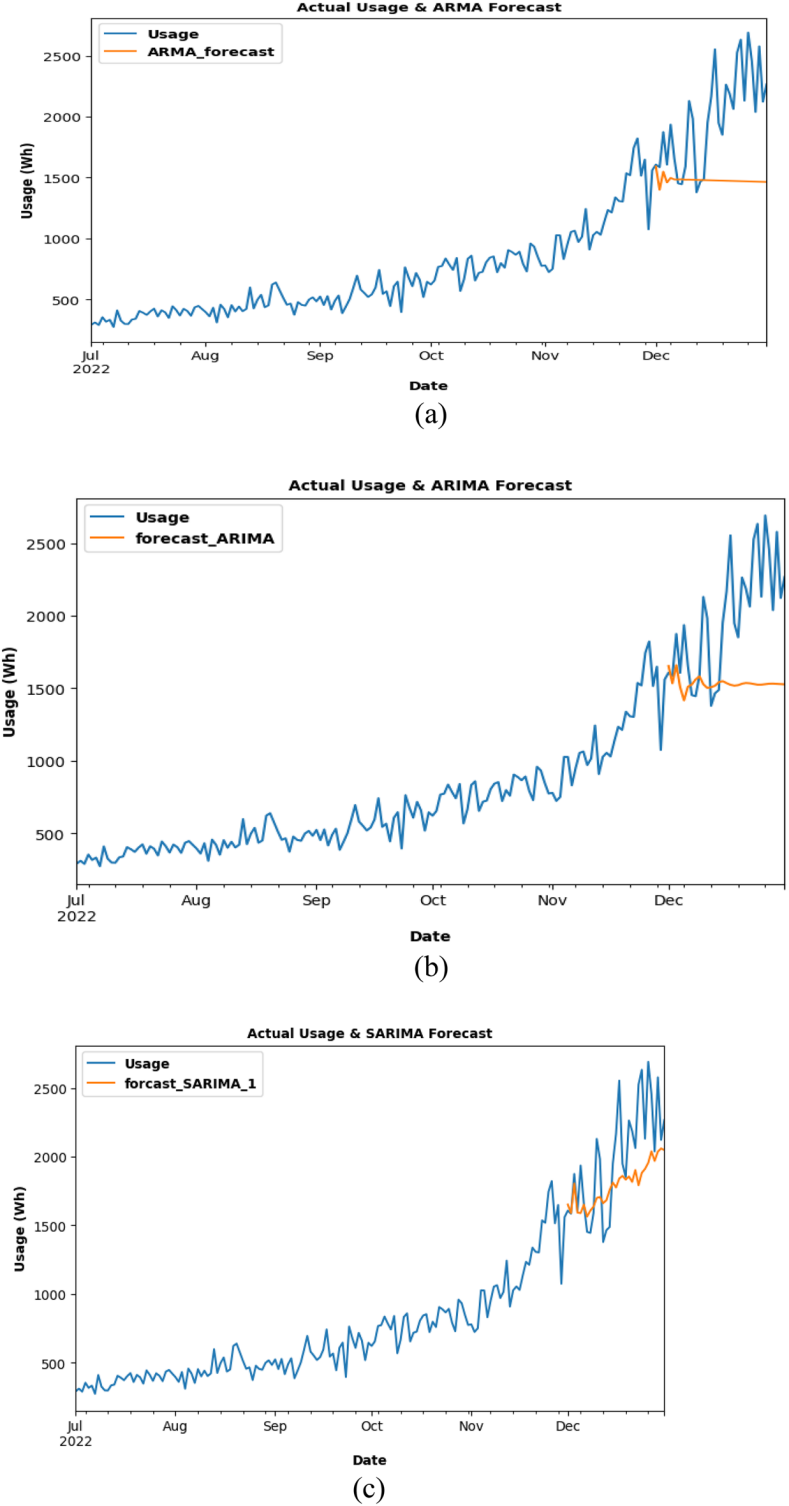
Charging activity for ARMA, ARIMA and SARIMA: (**a**) ARMA model testing (**b**) ARIMA model testing (**c**) SARIMA model testing for Charging Activity dataset.

The energy usage is forecasted using the ARMA model for the last 1 month for already existing data as in Fig. 14a and b, energy usage is forecasted using the ARIMA model for the last 1 month for already existing data. In Fig. 14c, energy usage is forecasted using the SARIMA model for the last 1 month for already existing data.

**Figure.15:**
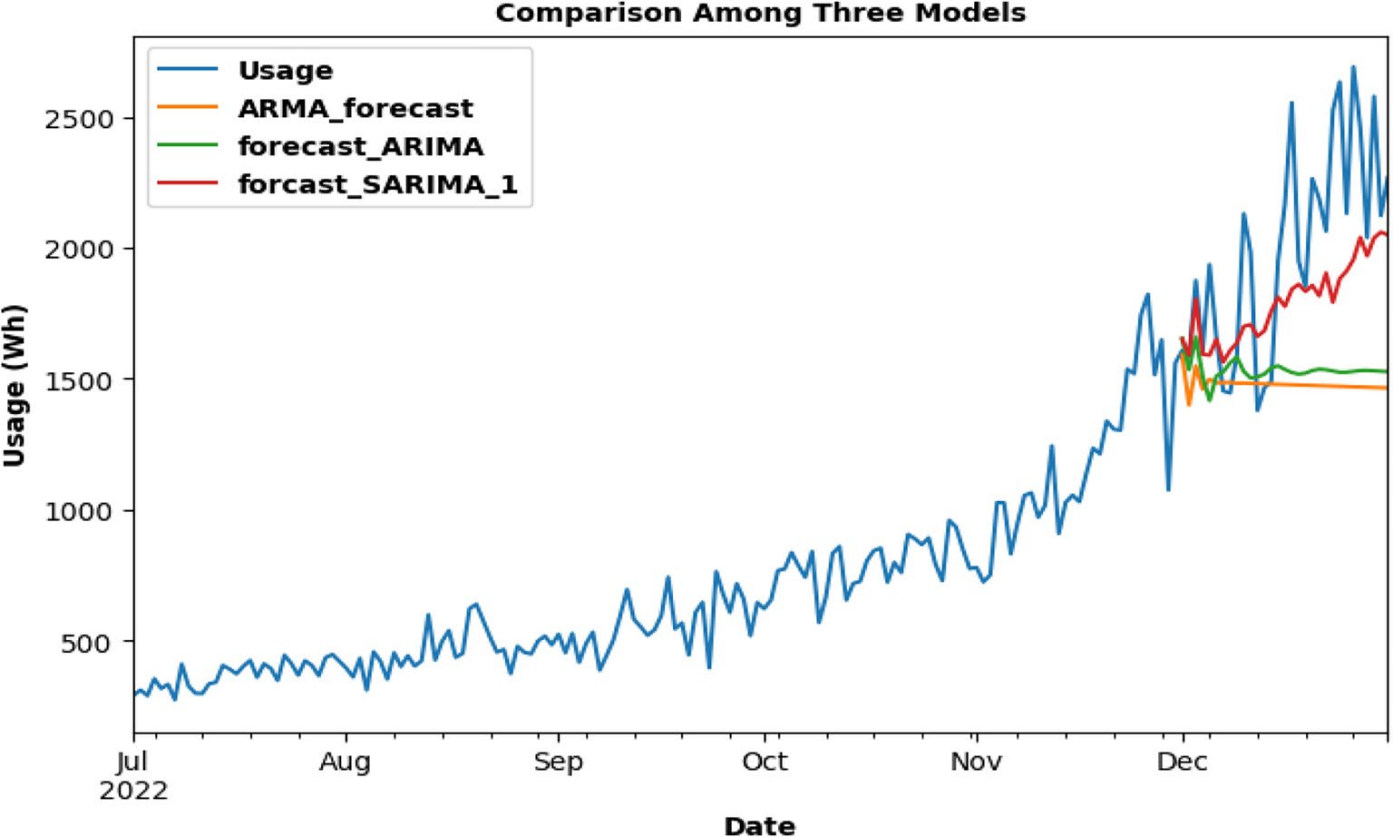
Comparison of 3 models.

**Figure. 16:**
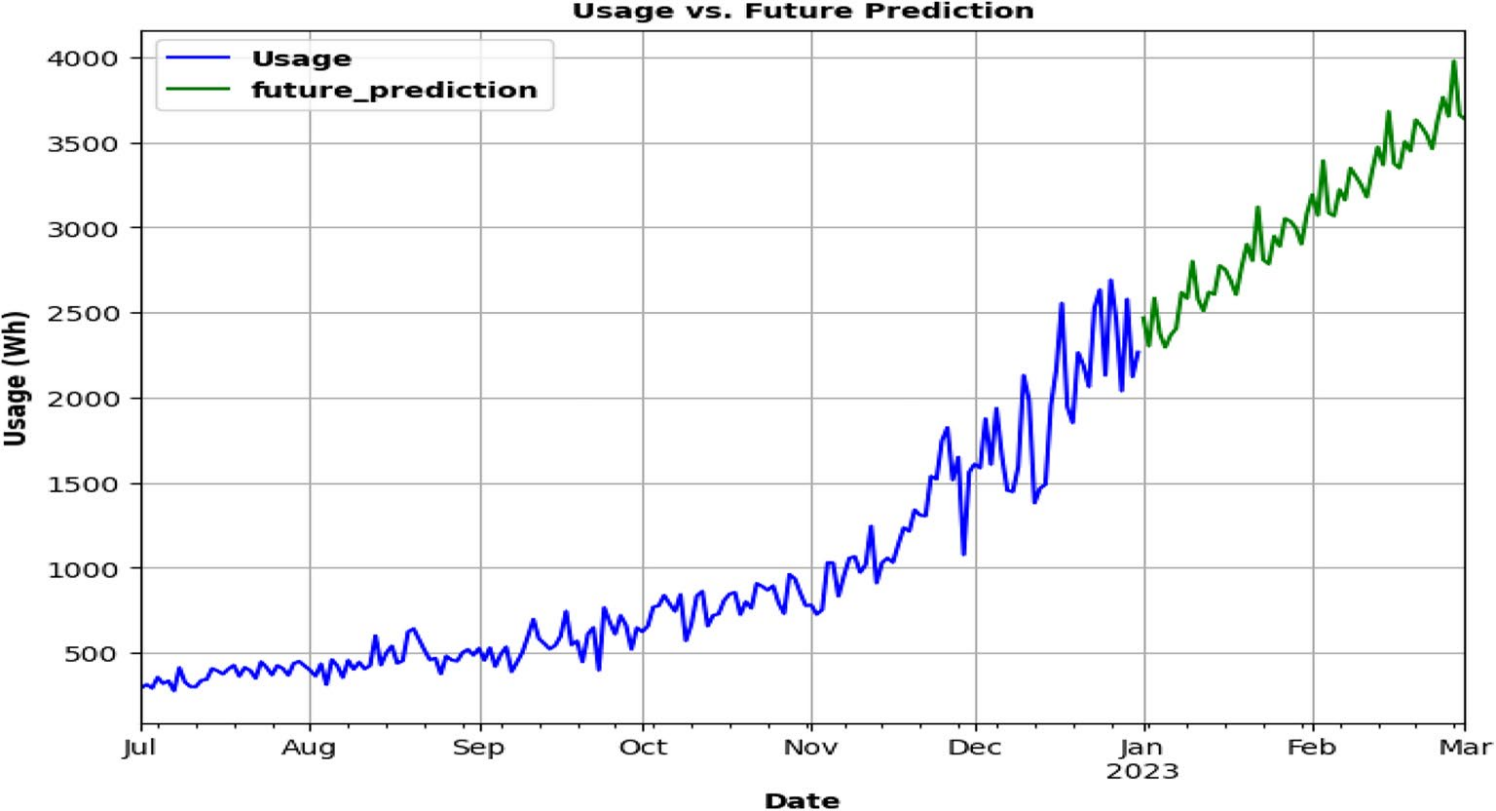
Future prediction : SARIMA model for 2 months.

Each model prediction is plotted in the same graph and accuracy is compared as seen from Fig. 15. In Fig. 16, the SARIMA model is being used for future usage consumption prediction of the Charging Activity dataset for 2 months. Upon analyzing the data, it is found that the power consumption varied significantly across different charging stations and time periods. it is also observed that the type of charging station (AC or DC) and the type of vehicle (two-wheeler, three-wheeler, or four-wheeler) had a significant impact on energy consumption.

Further analysis showed that the power consumption is higher in DC charging stations compared to AC charging stations, as expected. Additionally, it is found that four-wheelers consumed more electricity than two- and three-wheelers, which could be attributed to their larger size and higher power requirements. It also observed a trend of increasing energy consumption over time, which could be due to the growing popularity of electric vehicles and the increasing number of charging stations. Overall, these insights can be valuable for developing the necessary electrical infrastructure and evaluating the cost of operating EVs.

### Forecasting plots of subscription dataset

**Figure 17:**
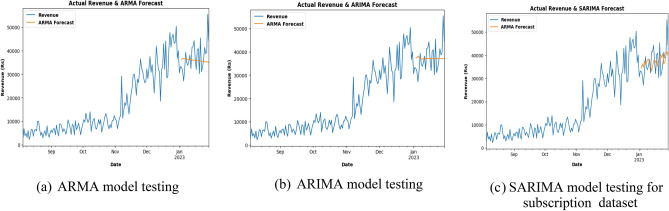
Subscription for ARMA, ARIMA and SARIMA.

The revenue from different subs plans is forecasted using ARMA model for last 1 month for already existing data is shown in Fig. 17a and b helps us to check whether the revenue from different subs plans is forecasted using ARIMA model for last 1 month for already existing data. After training and testing the ARMA model on the company plan subscription dataset, it became apparent that the graph is declining and the prediction is quite inaccurate, failing to capture the trend. The resulting RMSE, MAE, and MAPE scores are 6306.42, 5045.66, and 0.13, respectively. Although the ARIMA model showed a slight increase in prediction accuracy compared to the ARMA model, it still had a linear graph. The resulting RMSE, MAE, and MAPE scores are lower than those of the ARMA model. However, when the SARIMA model is applied, the results showed a better prediction accuracy than both the ARMA and ARIMA models, with a graph that is trending upwards, in line with the actual data.

Furthermore, the resulting RMSE, MAE, and MAPE scores are 5197.89, 4194.04, and 0.12, respectively, which are lower than the scores of the other models. Therefore, the SARIMA model is selected for future revenue predictions based on plan subscriptions for the company. It is forecasted that the company would generate a revenue of Rs. 66,853 by March 29th, 2023. Figure 17c, revenue from different subs plans is forecasted using the SARIMA model for the last 1 month for already existing data. A summary of the evaluation metrics for the subscription data set is mentioned in Table 4.

**Table 4:** Summar—evaluation metrics for subscription dataset.

MODEL	ARMA	ARIMA	SARIMA
RSME	6306.42	5954.49	5197.89
MAE	5045.66	4873.99	4194.04
MAPE	0.13	0.13	0.12

**Figure 18:**
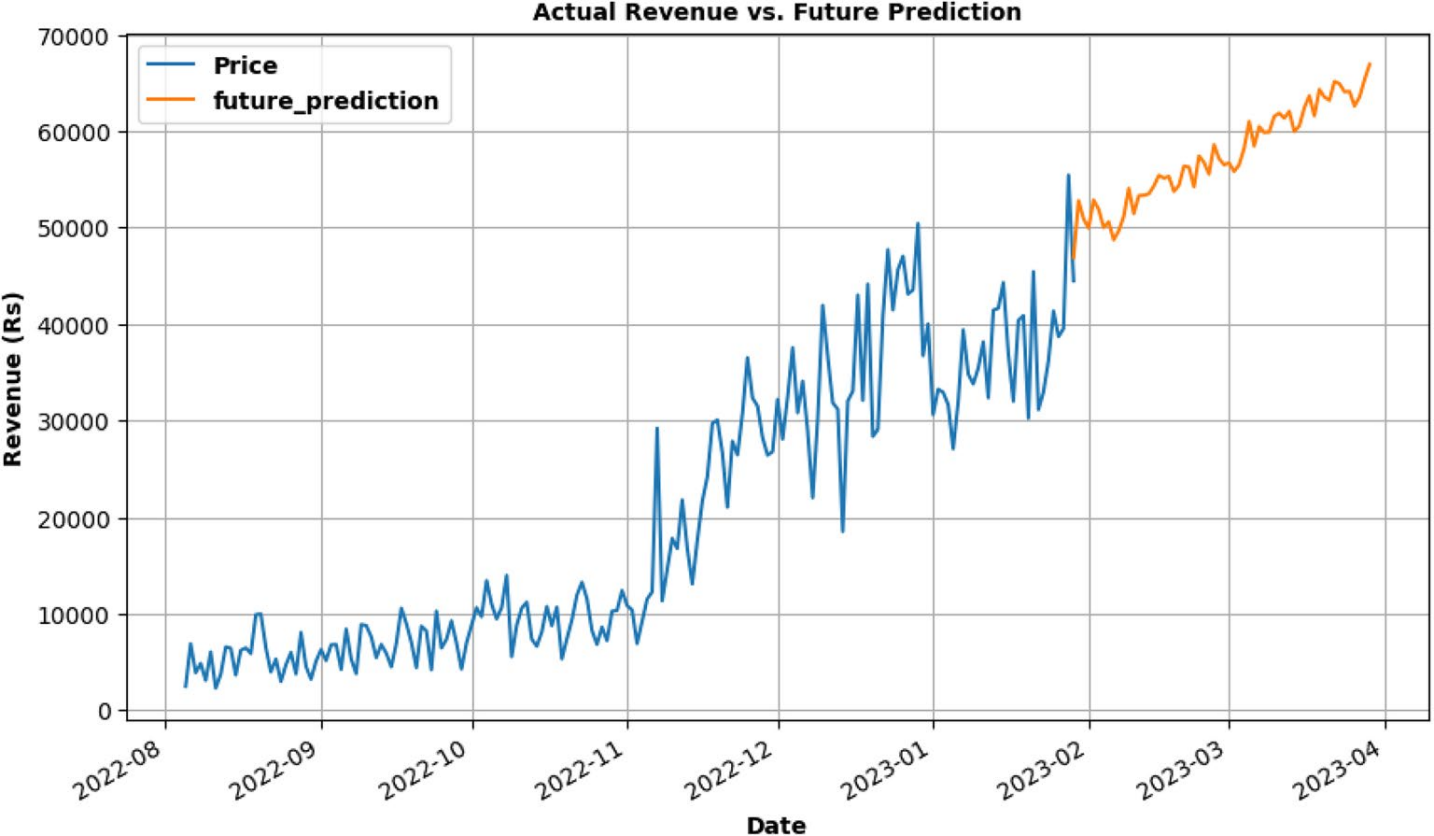
Future revenue prediction using SARIMA model for 2 months.

SARIMA model in Fig. 18 is being used for future revenue prediction of subs dataset for 2 months. Upon analyzing the data, it is found that plan Green and Rainbow plan as in Fig. 11 by generating significantly more revenue than others. It is also found that there is a strong correlation between the price of the subscription plan and the revenue generated. Additionally, it is observed that the revenue generated varied across different time periods, with some months generating more revenue than others. Overall, these findings can be useful in developing strategies to increase revenue for the company. By identifying the subscription plans that generate the most revenue, the company can focus on promoting those plans and optimizing their pricing. Additionally, analyzing the revenue trends across different time periods can help the company to better understand the demand for EV charging services and plan accordingly.

### Forecasting plots of AC and DC charging stations charging activity dataset

**Figure 19:**
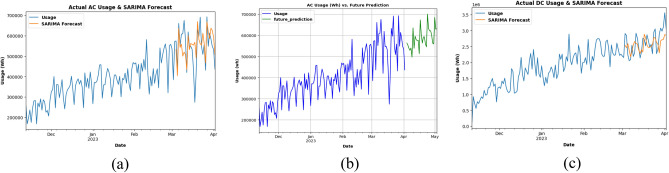
(**a**) SARIMA model testing for AC charging stations Charging Activity (**b**) Future prediction using SARIMA model for 1 month (**c**) SARIMA model testing for DC charging stations charging activity.

Energy usage for AC charging stations is forecasted in Fig. 19a using the SARIMA model for the last 2 months for already existing data and Fig. 19b uses SARIMA model for future consumption prediction of the AC charging station dataset for 1 month. EV charging stations that use AC (alternating current) are often used, and they take a lot of energy. Several variables, such as the charging rate, battery size, and charging duration, have an impact on how much energy AC charging stations use. The charging time for an EV depends on its battery capacity and is generally determined by the charging rate, which ranges from 3.3 to 22 kW at AC charging stations.

The time needed for charging increases with battery capacity. Although it has been noted that AC charging stations use less energy than DC charging stations, they nevertheless make a sizable contribution to the total energy usage. This is especially true when many EVs are being charged at once during rush hour. Installing more AC charging stations in areas where they are most required would help to reduce energy usage and the burden on the grid during peak hours. In Fig. 19c, energy usage for DC charging stations are forecasted using SARIMA model for last 2 month for already existing data.

**Figure. 20:**
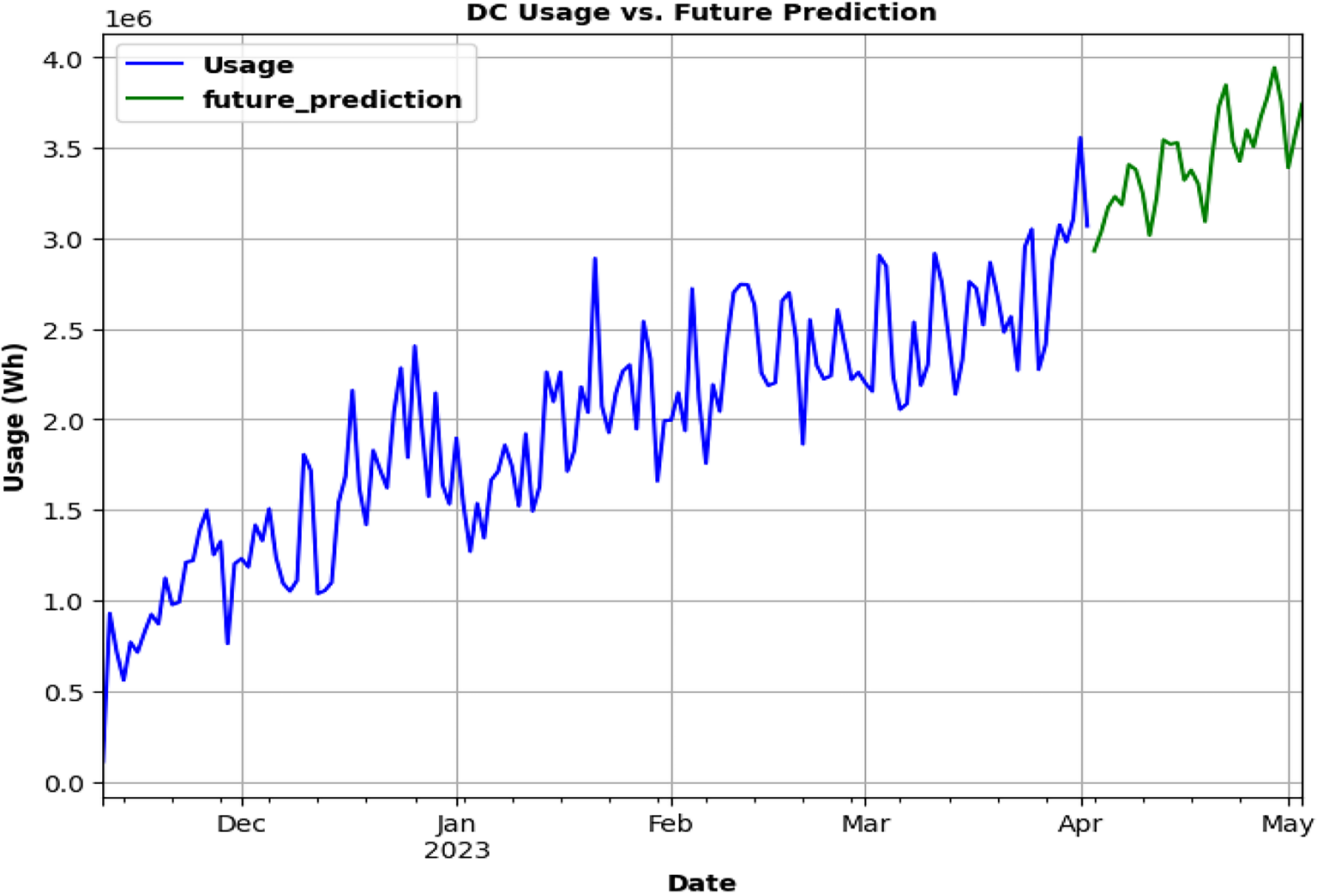
Future prediction using SARIMA model for 1 month.

SARIMA model is being used as in Fig. 20, for future usage consumption prediction of DC charging station dataset for 1 month. Upon analyzing the data, it is found that the power consumption is significantly higher in DC charging stations than in AC charging stations Fig. 19a and c. This can be attributed to the fact that DC charging stations typically have a higher power output, hence they can charge an EV more quickly. It is also observed that the energy consumption varied significantly across different charging stations, regardless of whether they are AC or DC stations.

### Forecasting plots of two-wheelers, three-wheelers and four-wheelers charging activity

**Figure.21:**
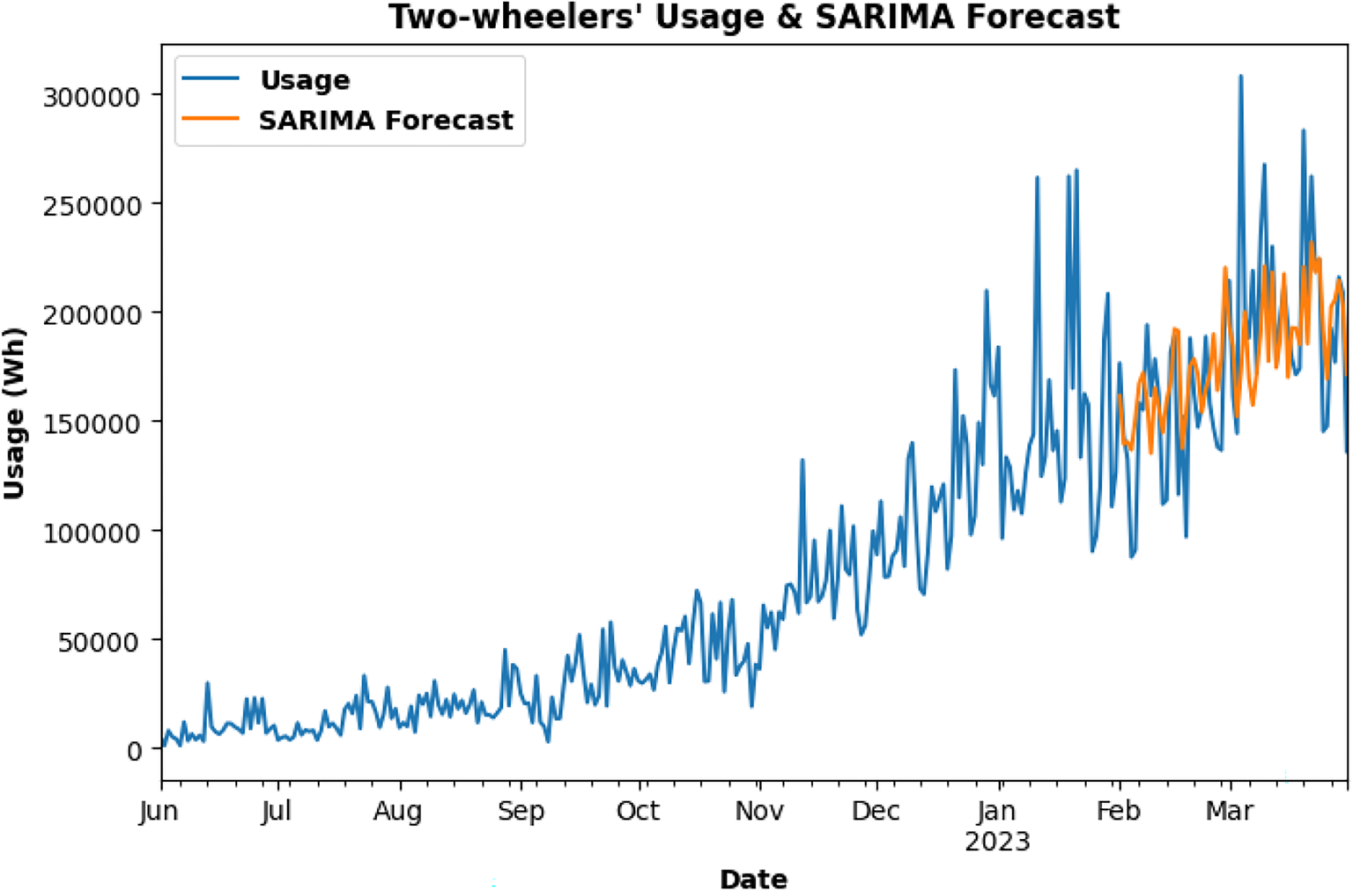
SARIMA model testing for Two-Wheelers Charging Activity.

**Figure. 22:**
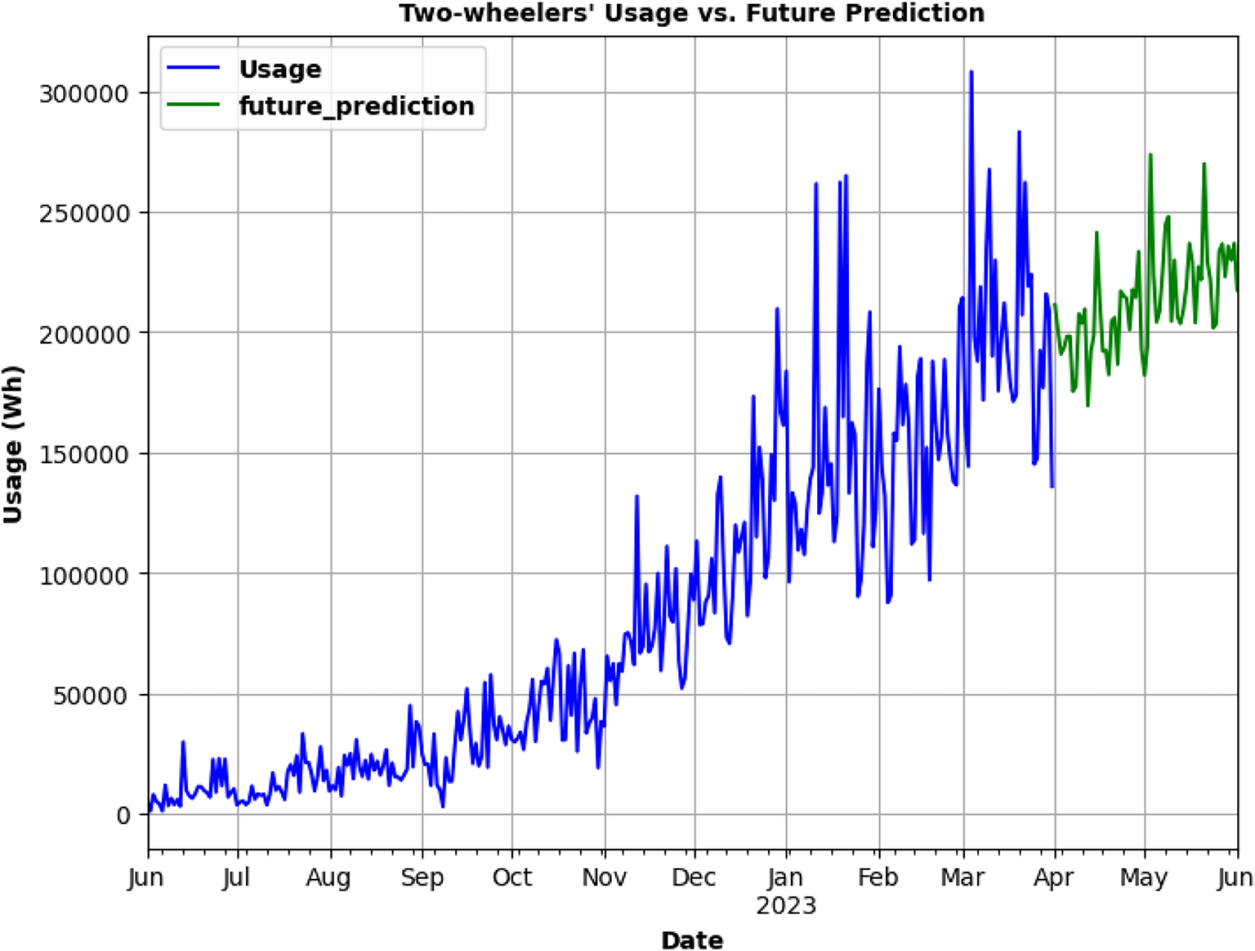
Future prediction using SARIMA model for 2 months.

Energy usage for Two-Wheelers is forecasted using the SARIMA model for the last 2 months for already existing data as shown in Fig. 21. In Fig. 22, the SARIMA model is being used for Two-Wheeler's future energy usage consumption for 2 months.

**Figure. 23:**
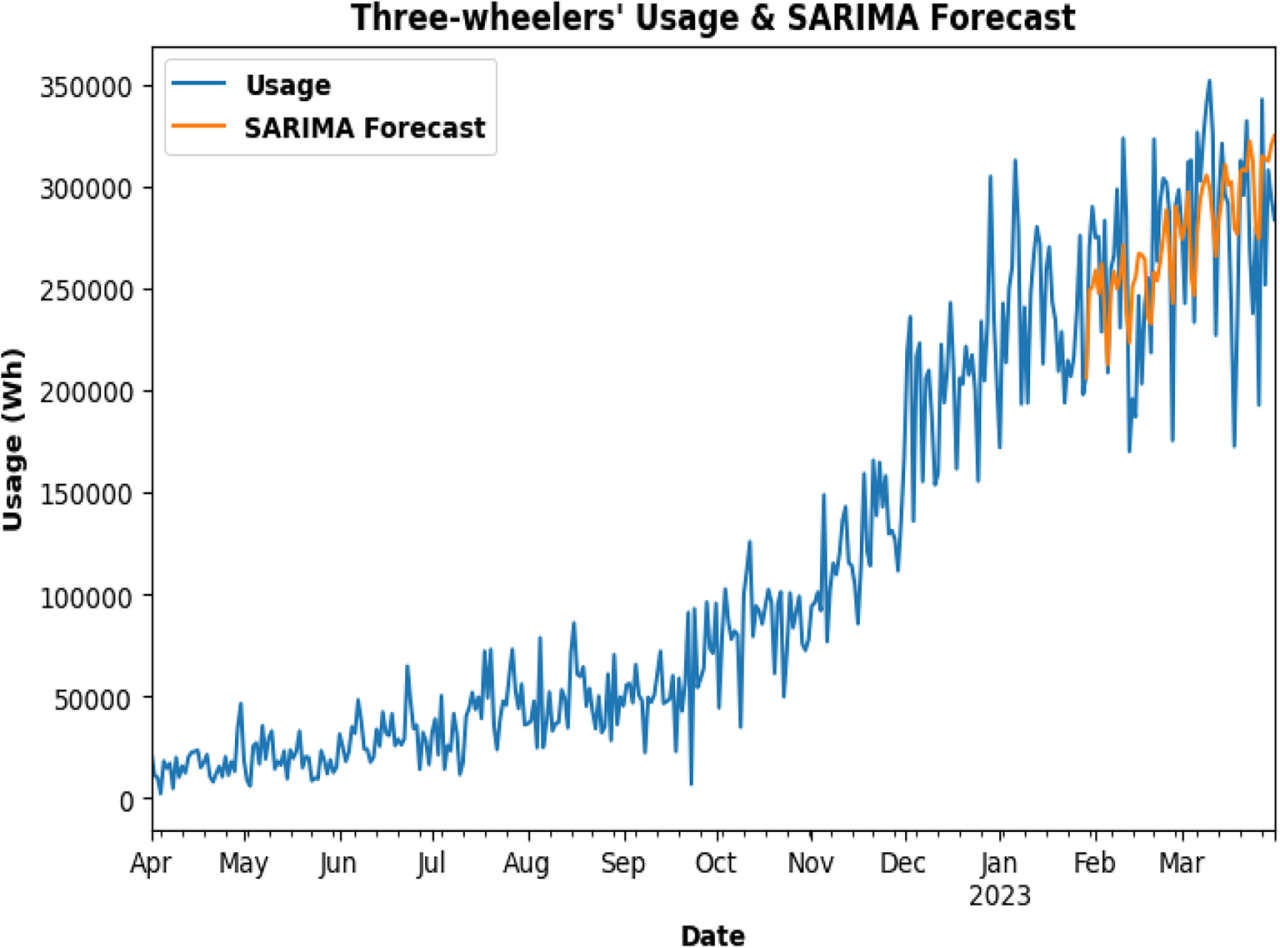
SARIMA model testing for Three-Wheelers' CA.

**Figure. 24:**
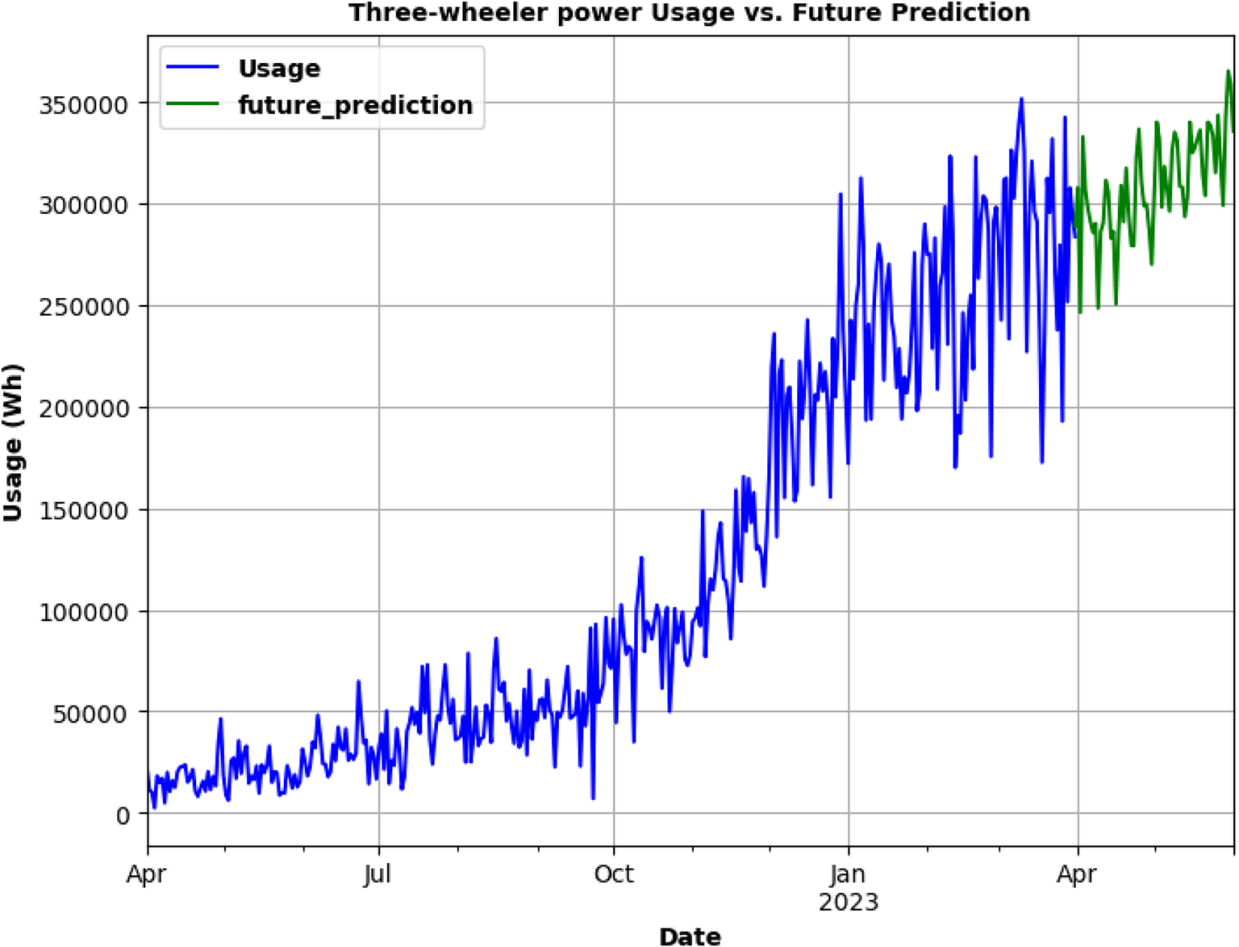
Future prediction using SARIMA model for 2 months.

**Figure 25:**
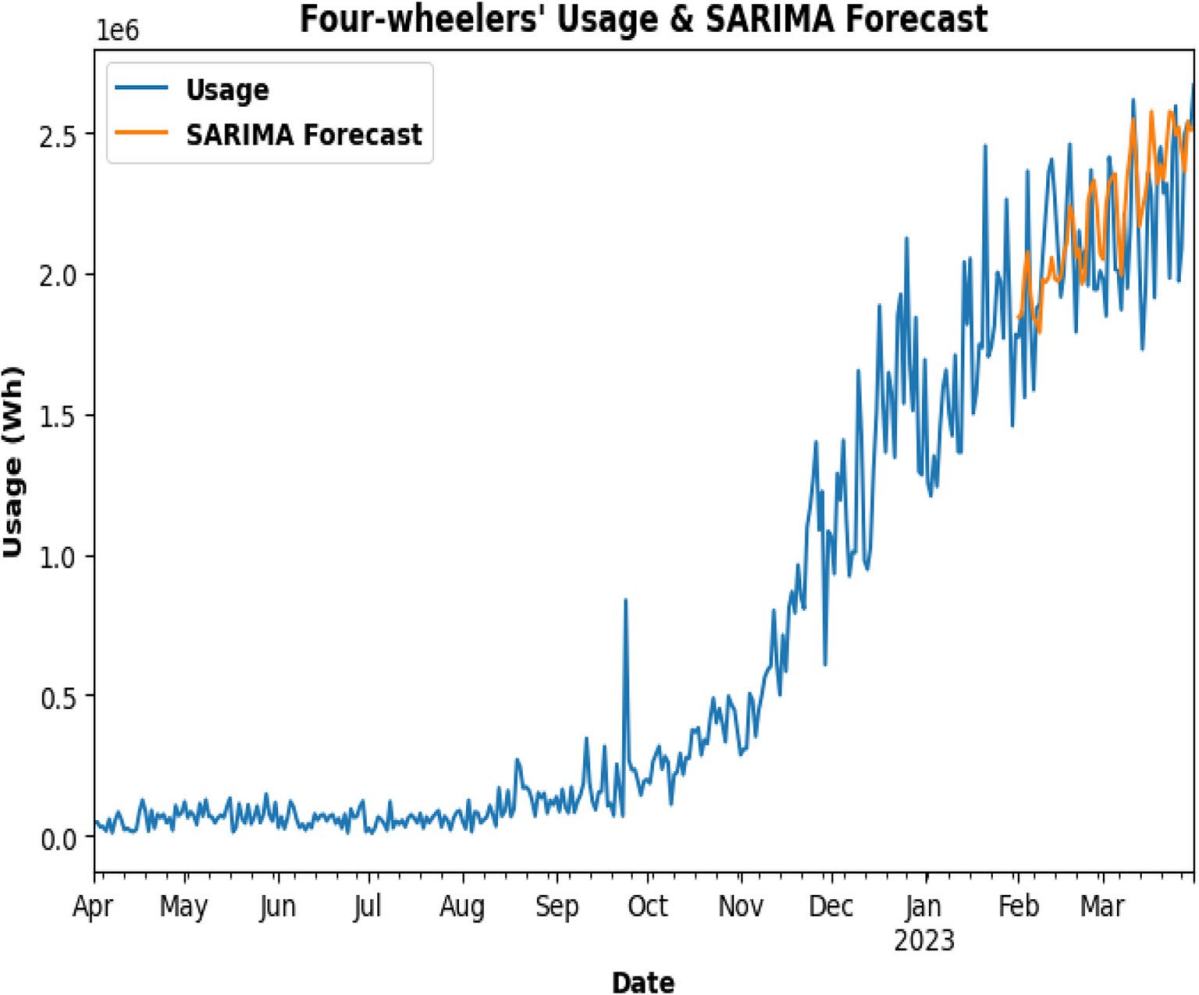
SARIMA model testing for four-wheelers charging activity dataset.

**Figure 26:**
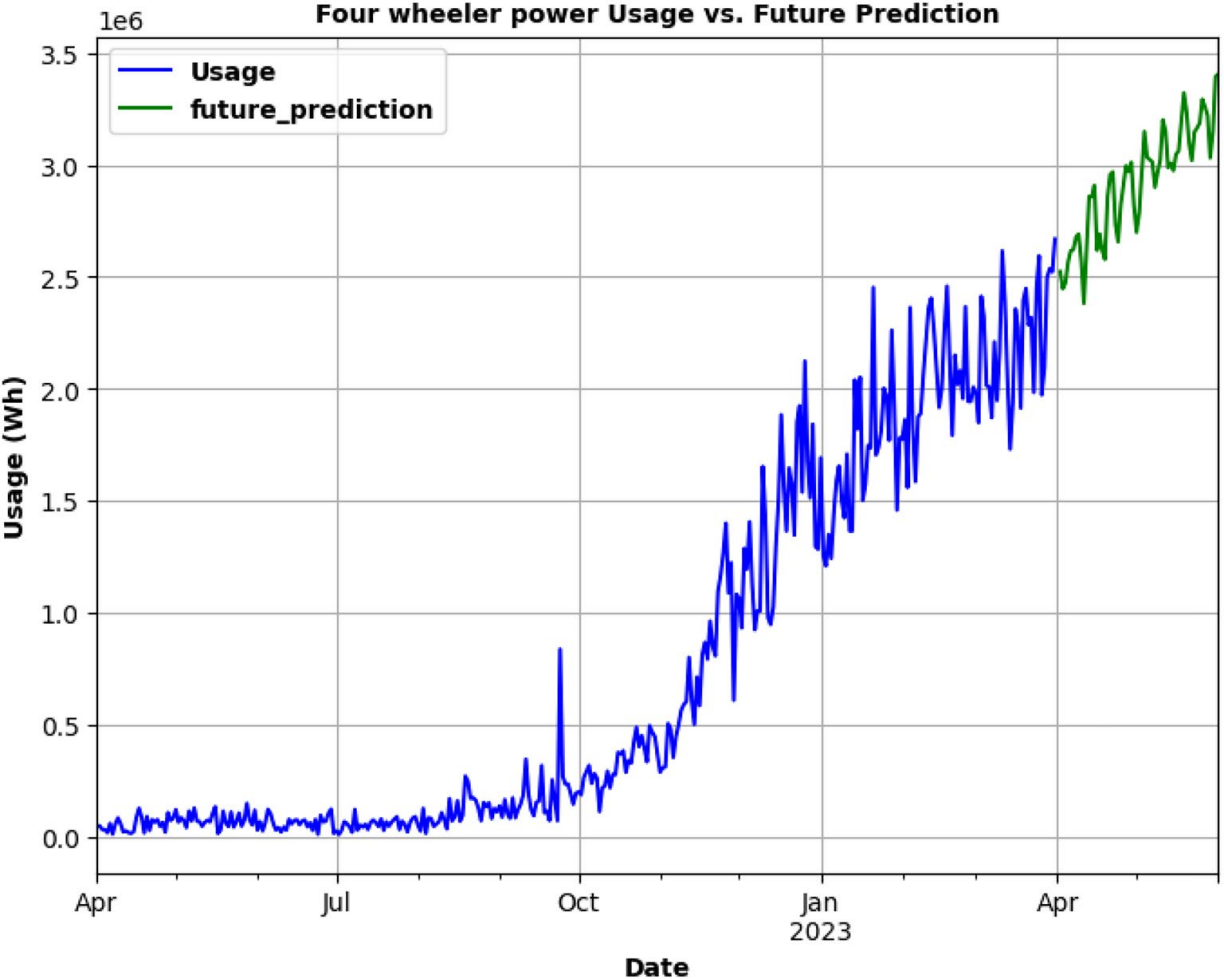
Future prediction using SARIMA model for 2 months.

SARIMA model is being used for testing as in Fig. 23 and to predict the future energy consumption of Three-Wheelers. In Fig. 24, SARIMA model is used for Three-Wheeler's future energy usage consumption for 2 months. SARIMA model is used for testing and to predict the future energy consumption of Four-Wheelers as can be seen in Fig. 25. In Fig. 26, SARIMA model is being used for Four-Wheeler's future energy usage consumption for 2 months. After analyzing the charging activity dataset of EVs based on vehicle types, it is found that the power consumption of four-wheelers is significantly higher compared to that of two-wheelers and three-wheelers. As four-wheelers have higher battery capacity and require more power for charging, the charging duration for four-wheelers is longer compared to that of two-wheelers and three-wheelers. It is also observed that the power consumption and charging duration of two-wheelers and three-wheelers are similar. This indicates that the charging infrastructure for two-wheelers and three-wheelers can be similar and can be optimized for faster charging. Overall, the analysis provides useful insights for developing necessary electrical infrastructure and evaluating the cost of operating EVs based on the vehicle type.

For power consumption prediction, time-series models including ARMA, ARIMA, and SARIMA are employed^40^. However, since the SARIMA model produced more accurate results as can be seen from Tables 1 and 2, than the other models, it was used for future power consumption prediction in all cases. Finally, a plan subscription dataset was obtained from chargeMOD, which contained plan subscription details of users, including the plan price. The SARIMA model was again used for forecasting revenue for the company through different plans.

## Conclusions

The results of our experiment show that the SARIMA model is the best in foretelling power usage for all three datasets. In particular, our forecasts indicated that on March 1, 2023, electricity consumption in Colorado is anticipated to be 17,765 (kWh), whereas it is predicted to be just 120.20 (kWh) in Kerala ChargeMOD. In addition, four-wheelers use more power than two- and three-wheelers, according to our study of the data. DC charging facilities also use more electricity than AC charging stations. The creation of crucial electrical infrastructure for electric car charging stations can benefit greatly from these revelations. Additionally, the results of our study may be used to determine how much it will cost to operate EV and to develop charging station and subscription pricing strategies. Overall, our initiative offers useful information on the power usage habits of EV charging stations and can help stakeholders build an EV infrastructure that is more efficient and sustainable.

First, as a way to increase the precision of the power consumption forecast, the potential for including additional real-time energy usage data is noted. By doing this, it could be feasible to expand these forecasts to specific stations and car models, giving consumers information that is more precise and focused. It becomes possible to develop and design charging guns that are tailored to the unique needs and specifications of various segments and models, ultimately improving the overall charging experience. Furthermore, it is recommended that further research be done on the effects of various price structures on consumer behavior. Companies may be able to improve their pricing strategies to better suit consumer demands and increase profitability by examining how this pricing plans influence revenue earned through charging stations. Charging stations may lessen their reliance on non-renewable energy sources by using the power of solar or wind turbines, eventually lowering the carbon footprint associated with charging Evs. Overall, the ideas presented lays the groundwork for further research and development in the field of EVCSs. By exploring these areas in more depth, the usability and profitability of EVCSs can be improved ultimately benefiting both users and businesses alike.

### Supplementary Information

Supplementary Information.

## Data Availability

The Colorado datasets analysed during the current study are available in the [Colorado dataset] repository, https://open-data.bouldercolorado.gov/datasets/95992b3938be4622b07f0b05eba95d4c which is used for energy consumption. The dataset 2 and 3 that support the findings of this study are available from [chargeMOD] but restrictions apply to the availability of these data, which were used under license for the current study, and so are not publicly available. Data are however available from the authors upon reasonable request and with permission of [chargeMOD]. hello@chargemod.com.
