# Supplementary material for: Power consumption prediction for electric vehicle charging stations and forecasting income
Source: Sci Rep. 2024 Mar 18;14:6497. doi: 10.1038/s41598-024-56507-2 (PMC10948759; doi:10.1038/s41598-024-56507-2)

Power Consumption Prediction for Electric Vehicle Charging Stations and Forecasting Income

Akshay K C ^1^, Hannah Grace G^1*^ , Kanimozhi Gunasekaran ^2*^ , Ravi Samikannu^3^

^1^ School of Advanced Sciences, Vellore Institute of Technology, Chennai, Tamil Nadu, India. Email: hannahgrace.g@vit.ac.in

^2^ Center for Smart Grid Technologies, School of Electrical Engineering, Vellore Institute of Technology, Chennai, Tamil Nadu, India. Email: Kanimozhi.g@vit.ac.in

^3^ Botswana International University of Science and Technology, Palapye, Botswana.

Three data sets are used in this paper

1. **COLORADO Dataset**
2. **Charging Activity Dataset**
3. **Subscription Dataset**

**COLORADO DATASET DESCRIPTION – 1**

The datasets generated and/or analysed during the current study are available in the [Colorado dataset] repository, <https://open-data.bouldercolorado.gov/datasets/95992b3938be4622b07f0b05eba95d4c>

which is used for energy consumption.

The Colorado Energy Consumption dataset is a data set that provides information on the energy consumption patterns in the state of Colorado, United States. The attributes in the EV charging station dataset provide information about the location, usage, and environmental impact of EV charging stations. The attributes include the station name, address, city, state or province, postal code, and type of charging port used. Additionally, the data includes the start and end dates and times of charging sessions, the total duration and charging time, and the amount of energy consumed in kilowatt-hours, as well as the estimated greenhouse gas emissions saved and equivalent gasoline consumption.

**SAMPLE DATA SET -1 ( COLORADO Data set)**


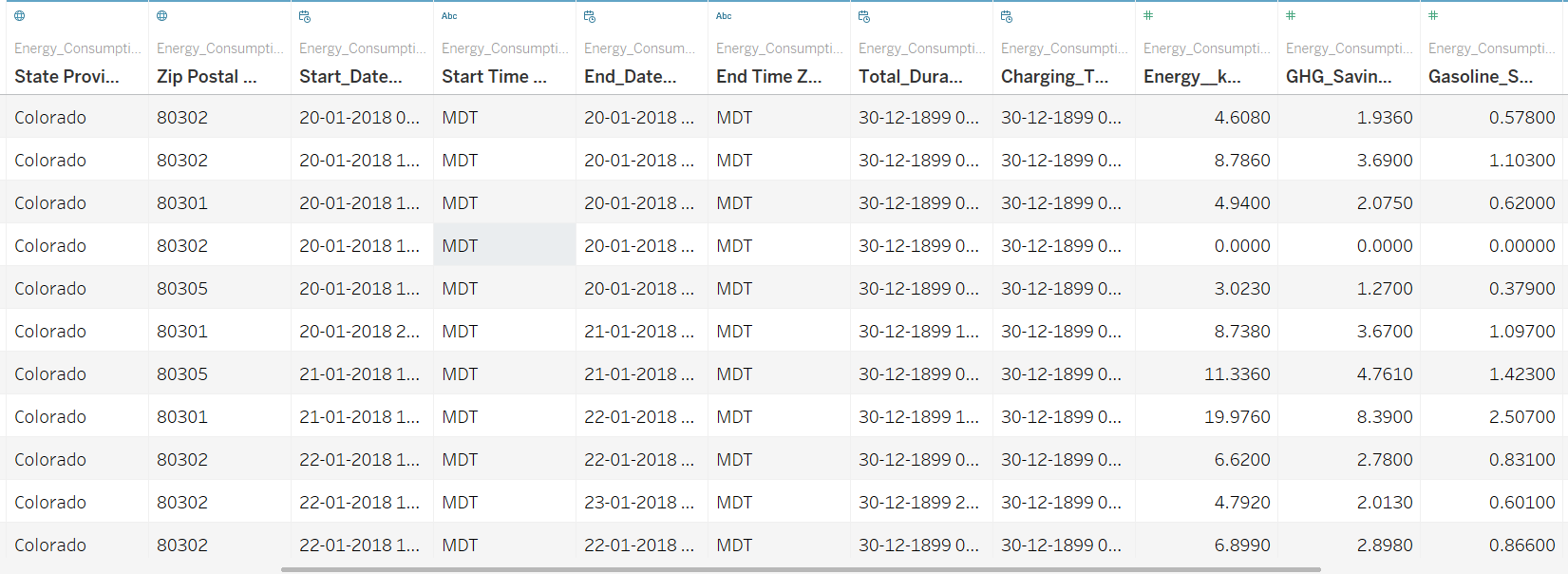


**Description about Dataset 2 & 3**

The data that support the findings of this study are available from [chargeMOD] but restrictions apply to the availability of these data, which were used under license for the current study, and so are not publicly available. Data are however available from the authors upon reasonable request and with permission of [chargeMOD].

**Acknowledgement:**


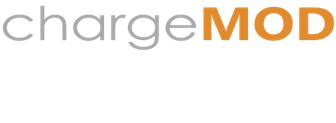


BPM POWER PRIVATE LIMITED (chargeMOD) is startup in found in 2019. ​It specializes in providing electric vehicle charging services. ​It also provides other services through app like locating nearest charging station, monitoring charging sessions etc. ​ ChargeMOD provides the customers with a variety of charging subscription plans. ​It has charging stations in all districts of Kerala. Chennai, Ooty, Pune and Bangalore round out the list. ​ With networked Smart Station ChargeMOD makes charging fast, simple and safe. ​The future motive of the company is to provide all Electric Vehicle services to the customers.​

**CHARGING ACTIVITY DATASET DESCRIPTION – 2**

The Charging Activity dataset from chargeMOD is a dataset that provides information on the energy consumption patterns mainly in the state of Kerala, India. Mainly it contains information about electric vehicle charging sessions, including the user who used the charging station, the charging station itself, and the characteristics of the charging session (e.g. start and stop times, amount of energy used). This data could be used to analyze patterns of electric vehicle usage, understand the performance of different charging stations, and inform decisions about the installation of new charging infrastructure.

**SAMPLE DATASET - 2**


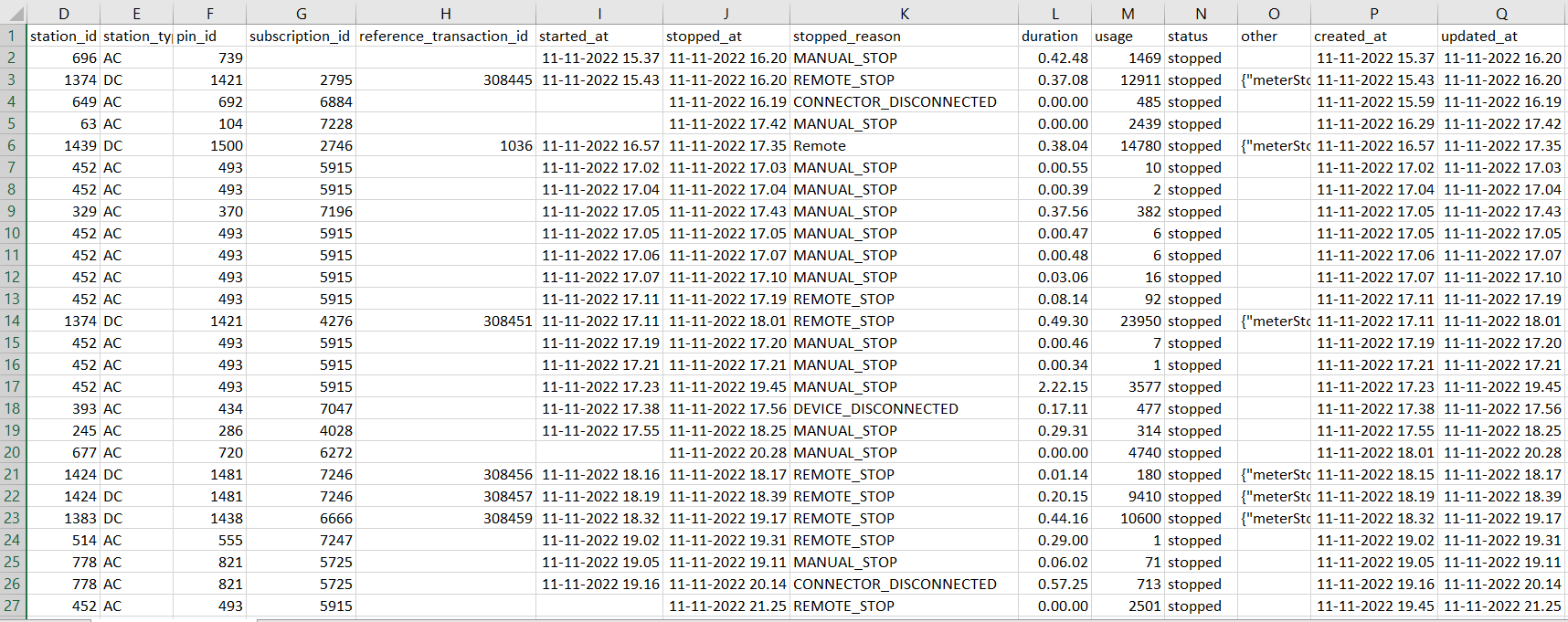


**Subscription Dataset Description – 3**

The dataset contains information related to user subscription plans and payment details.

- id: A unique identifier for each record in the dataset.
- user_id: An identifier for the user who has subscribed to a particular plan.
- plan_id: An identifier for the subscription plan.
- payment_id: An identifier for the payment transaction.
- plan_name: The name of the subscription plan.
- plan_price: The price of the subscription plan.
- plan_duration: The duration of the subscription plan.
- plan_energy: The amount of energy provided by the subscription plan in (Wh).
- balance: The current balance energy in user's account in (Wh).
- status: The status of the subscription plan, such as active, expired, or canceled.
- executed_at: The date and time when the subscription plan was executed.
- expire_at: The date and time when the subscription plan will expire.
- payment_status: The status of the payment transaction, such as successful, failed, or pending.
- created_at: The date and time when the record was created.
- updated_at: The date and time when the record was last updated.

**SAMPLE DATASET - 3**


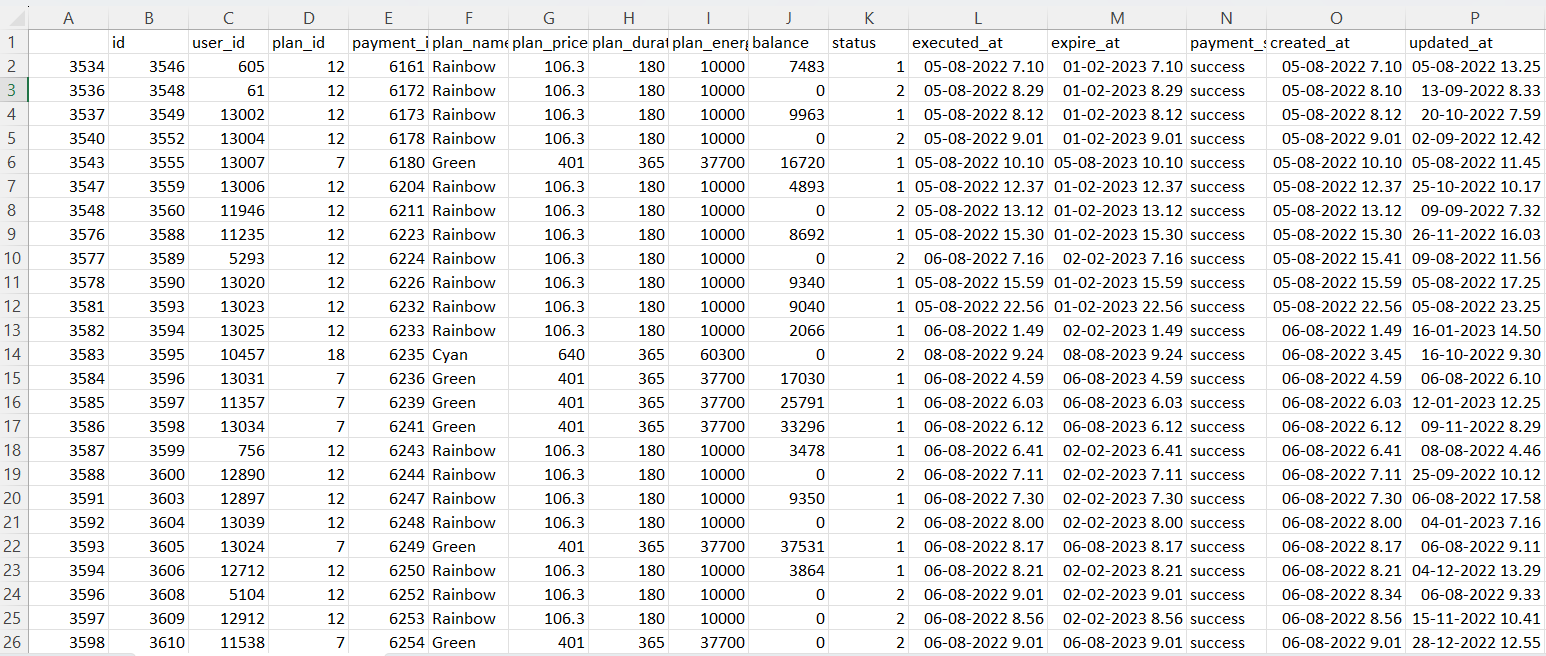

Supplement: Supplementary file 1 — Supplementary Information. [file 41598_2024_56507_MOESM1_ESM.docx]
